# Limitations and tradeoffs in synchronization of large-scale networks with uncertain links

**DOI:** 10.1038/srep21157

**Published:** 2016-04-12

**Authors:** Amit Diwadkar, Umesh Vaidya

**Affiliations:** 1 Electrical and Computer Engineering, Iowa State University Coover Hall, Ames, IA, USA 50011

## Abstract

The synchronization of nonlinear systems connected over large-scale networks has gained popularity in a variety of applications, such as power grids, sensor networks, and biology. Stochastic uncertainty in the interconnections is a ubiquitous phenomenon observed in these physical and biological networks. We provide a size-independent network sufficient condition for the synchronization of scalar nonlinear systems with stochastic linear interactions over large-scale networks. This sufficient condition, expressed in terms of nonlinear dynamics, the Laplacian eigenvalues of the nominal interconnections, and the variance and location of the stochastic uncertainty, allows us to define a synchronization margin. We provide an analytical characterization of important trade-offs between the internal nonlinear dynamics, network topology, and uncertainty in synchronization. For nearest neighbour networks, the existence of an optimal number of neighbours with a maximum synchronization margin is demonstrated. An analytical formula for the optimal gain that produces the maximum synchronization margin allows us to compare the synchronization properties of various complex network topologies.

Synchronization in large-scale network systems is a fascinating problem that has attracted the attention of researchers in a variety of scientific and engineering disciplines. It is a ubiquitous phenomenon in many engineering and naturally occurring systems, with examples including generators for electric power grids, communication networks, sensor networks, circadian clocks, neural networks in the visual cortex, biological applications, and the synchronization of fireflies^1^,^2^,^3^,^4^. The synchronization of systems over a network is becoming increasingly important in power system dynamics. Simplified power system models demonstrating synchronization are being studied to gain insight into the effect of network topology on the synchronization properties of dynamic power networks^5^. The effects of network topology and size on the synchronization ability of complex networks is an important area of research^6^. Complex networks with certain desirable properties, such as a small average path between nodes, low clustering ability, and the existence of hub nodes, among others, have been extensively studied over the past decade^7^,^8^,^9^,^10^,^11^,^12^.

It is impossible to do justice to the long list of literature that exists in the area of synchronization of dynamical systems. In the following discussion, we list a few references that are particularly relevant to the results presented in this paper. In^13^, the master stability function was introduced to study the local synchronization of chaotic oscillator systems. Interesting computational observations were made that indicated the importance of the smallest and largest eigenvalues of the graph Laplacian. The master stability function was also used to study synchronization over Small-World networks and provide bounds on the coupling gains to guarantee the stability of the synchronous state in^14^. Bounds were provided on the coupling gains to guarantee the stability of the synchronous state in^15^. The impact of network interconnections on the stability of the synchronous state of a network system was also studied in^16^. These results derived a condition for global synchronization based on the coupling weights and eventual dissipativity of the chaotic system using Lyapunov function methods and a bound on path lengths in the connection graph. In this paper, as in the papers listed above, we provide an analytical characterization of the importance of the smallest and largest positive eigenvalue of the coupling Laplacian. However, in contrast to the above references, we provide conditions for the global synchronization in the presence of stochastic link uncertainty. Understanding the role of spatial perturbation in the nearest neighbour network to force a transition from one synchronized state to another is important for molecular conformation^17^. Other aspects of network synchronization that are gaining attention are the effects of network topology and interconnection weights on the robustness of the synchronization properties^18^. In this paper, we provide a systematic approach for understanding the effects of stochastic spatial uncertainties, network topology, and coupling weights on network synchronization.

Uncertainty is ubiquitous in many of these large-scale network systems. Hence, the problem of synchronization in the presence of uncertainty is important for the design of robust network systems. The study of uncertainty in network systems can be motivated in various ways. For example, in electric power networks, uncertain parameters or the outage of transmission lines are possible sources of uncertainty. Similarly, a malicious attack on network links can be modelled as uncertainty. Synchronization with limited information or intermittent communication between individual agents, e.g., a network of neurons, can also be modelled using time-varying uncertainty. In this paper, we address the problem of robust synchronization in large-scale networks with stochastic uncertain links. Existing literature on this problem has focused on the use of Lyapunov function-based techniques to provide conditions for robust synchronization^19^.

Both the master stability function and Lyapunov exponents have been used to study the variation of the synchronous state’s stability, given local stability results with stochastic interactions^20^,^21^. The problem of synchronization in the presence of simple on-off or blinking interaction uncertainty was studied in^22^,^23^,^24^,^25^ using connection graph stability ideas^16^. The local synchronization of coupled maps was studied in^26^,^27^, which also provides a measure for local synchronization. Synchronization over balanced neuron networks with random synaptic interconnections has also been studied^28^. Researchers have studied the emergence of robust synchronized activity in networks with random interconnection weights^29^. The robustness of synchronization to small perturbations in system dynamics and noise has been studied^30^, while the robustness to parameter variations was also studied in the context of neuronal behaviour^31^. In this paper, we consider a more general model for stochastic link uncertainty than the simple blinking model and develop mathematically rigorous measures to capture the degree of synchronization.

We consider a network of systems where the nodes in the network are dynamic agents with scalar nonlinear dynamics. These agents are assumed to interact linearly with other agents or nodes through the network Laplacian. The interactions between the network nodes are assumed to be stochastic. This research builds on our past work, where we developed an analytical framework using system theoretic tools to understand the fundamental limitations of the stabilization and estimation of nonlinear systems with uncertain channels^32^,^33^,^34^,^35^. There are two main objectives for this research, which also constitute the main contributions of this paper. The first objective is to provide a scalable computational condition for the synchronization of large-scale network systems. We exploit the identical nature of the network agent dynamics to provide a sufficient condition for synchronization, which involves verifying a *scalar* inequality. This makes our synchronization condition independent of network size and hence computationally attractive for large-scale network systems. The second objective and contribution of this paper is to understand the *interplay* between three network characteristics: (1) internal agent dynamics, (2) network topology captured by the nominal graph Laplacian, and (3) uncertainty statistics in the network synchronization. We use tools from robust control theory to provide an analytical expression for the synchronization margin that involves all three network parameters and increases the understanding of the *trade-offs* between these characteristics and network synchronization. This analytical relationship provides useful insight and can compare the robustness properties for nearest neighbour networks with varying numbers of neighbours. In particular, we show that there exists an optimal number of neighbours in a nearest neighbour network that produces a maximum synchronization margin. If the number of neighbours is above or below this optimal value, then the margin for synchronization decreases.

We use an analytical expression for the optimal gain and synchronization margin to compare the synchronization properties of Small-World and Erdos-Renyi network topologies.

## Results

### Synchronization in Dynamic Networks with Uncertain Links

We consider the problem of synchronization in large-scale nonlinear network systems with the following scalar dynamics of the individual subsystems:
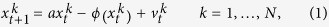
where 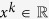 are the states of the 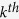 subsystem and 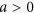 and 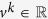 is an independent, identically distributed (i.i.d.) additive noise process with zero mean (i.e., 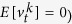 and variance 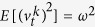. The subscript *t* used in Eq. (1) denotes the index of the discrete time-step throughout the paper. The function 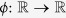 is a monotonic, globally Lipschitz function with 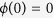 and Lipschitz constant 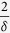 for *δ* > 0.

The individual subsystem model is general enough to include systems with steady-state dynamics that could be stable, oscillatory, or chaotic in nature. We assume the individual subsystems are linearly coupled over an undirected network given by a graph 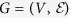 with node set *V*, edge set 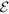, and edge weights 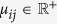 for 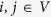 and 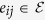. Let 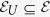 be a set of uncertain edges and 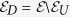. The weights for 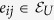 are random variables: 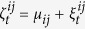, where 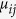 models the nominal edge weight and 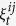 models the time-varying zero-mean uncertainty 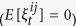, for all *t*, with known variance 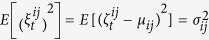, for all *t*. Because the network is undirected, the Laplacian for the network graph is symmetric. We denote the nominal graph Laplacian by 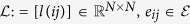, where 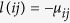, if 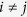, and, 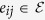, 
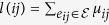
, if 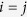. We denote the zero-mean uncertain graph Laplacian by 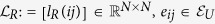, where 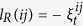, if 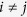, and, 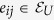, 
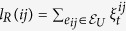
, if 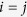. The nominal graph Laplacian *L* is a sum of the graph Laplacian for the purely deterministic graph 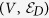, and of the mean Laplacian for the purely uncertain graph 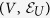. Hence, 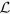 may be written as 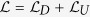, where 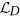, is the Laplacian for the graph over *V* with edge set 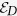. 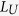 is the mean Laplacian for the graph over *V* with edge set 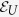. Define 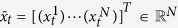 and 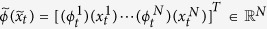, where 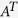 denotes the transpose of matrix *A*. In compact form, the network dynamics are written as
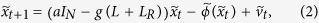
where *g* > 0 is the coupling gain and *I*_*N*_ is the 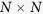 identity matrix. Our objective is to understand the interplay of the following network characteristics: the internal dynamics of the network components, the network topology, the uncertainty statistics, and the coupling gain for network synchronization. Given the stochastic nature of network systems, we propose the following definition of mean square synchronization^36^.

#### Mean Square Synchronization

Define 
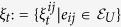
, 
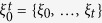
, 
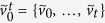
 and 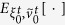 as the expectation with respect to uncertainties in the set 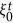 and 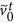. The network system (2) is said to be mean square synchronizing (MSS) if there exist positive constants 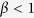, 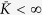, and 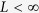, such that
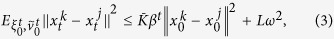

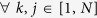
, where 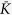 is a function of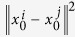 for 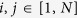 and 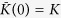 is a constant. In the absence of additive noise 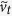 in system Eq. (2), the term 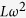 in Eq. (3) vanishes and the system is mean square exponential (MSE) synchronizing^37^. We introduce the notion of the *coefficient of dispersion* to capture the statistics of uncertainty.

#### Coefficient of Dispersion

Let 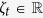 be a random variable with mean 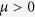 and variance 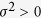. The coefficient of dispersion (CoD) *γ* is defined as 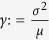. For all edges 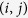 in the network, the mean weights assigned are positive, i.e., 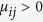 for all 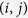. Furthermore, the CoD for each link is given by 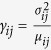 and 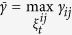.

Because the subsystems are identical, the synchronization manifold is spanned by the vector 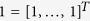. The dynamics on the synchronization manifold are decoupled from the dynamics off the manifold and are essentially described by the dynamics of the individual system, which could be stable, oscillatory, or complex in nature. We apply a change of coordinates to decompose the system dynamics on and off the synchronization manifold. Let 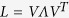, where *V* is an orthonormal set of vectors given by 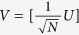, in which *U* is a set of 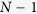 orthonormal vectors that are orthonormal to 1. Furthermore, we have 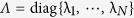, where 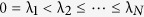 are the eigenvalues of 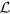. Let 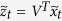 and 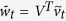. Multiplying (2) from the left by 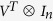, we obtain
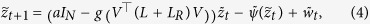
where 
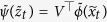
. We can now write 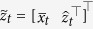, 
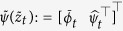
, and 
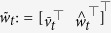
, where 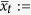 
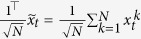
, 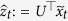, 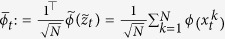, 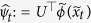, 
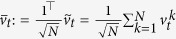
, and 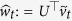. Furthermore, we have 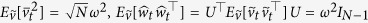 and 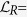

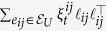
, where 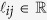 is 1 and −1 in the 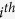 and 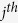 entries, respectively, and zero elsewhere. Thus, 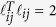 for all 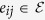. Hence, if 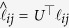, we have 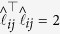 for all edges 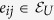 and 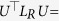 
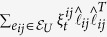
. From (4), we obtain 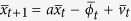 and
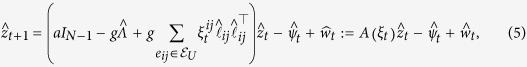
where 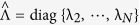 and 
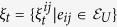
. For the synchronization of system (2), we only need to demonstrate the mean square stability about the origin of the 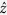 dynamics as given in (5).

The objective is to synchronize, in a mean square sense, *N* first-order systems over a network with a nominal graph Laplacian *L* with eigenvalues 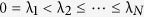 and maximum link CoD 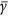. We present the main result of this paper.

#### Mean Square Synchronization Result

The network system in Eq. (2) is MSS if there exists a positive constant 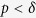 that satisfies
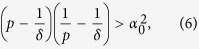
where 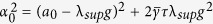, 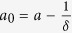 and 
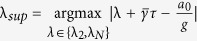
. Furthermore, 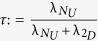, where 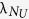 is the maximum eigenvalue of 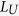 and 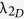 is the second-smallest eigenvalue of 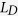.

The derivation of this result will be discussed in the Methods section. The above synchronization result relies on a Lyapunov function-based stability theorem. The positive constant *p* in Eq. (6) is used in the construction of the Lyapunov function given by 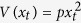. Furthermore, in the Methods section, we prove that the Mean Square Synchronization Result obtained in (6) is equivalent to
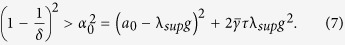


The main result can be interpreted in multiple ways. One particular interpretation useful in the subsequent definition of the synchronization margin is adapted from robust control theory. The robust control theory results allow one to analyse the stability of the feedback system with uncertainty in the feedback loop. The basic concept is that if the product of the system gain and the gain of the uncertainty (also called the loop gain) are less than one, then the feedback system is stable^38^. Note that system and uncertainty gains are measured by appropriate norms. The farther the system gain is from unity, the more uncertainty the feedback loop can tolerate and hence the more robust the system is to uncertainty. This result from robust control theory is extended to the case of stochastic uncertainty and nonlinear system dynamics^21^,^32^,^33^,^34^,^35^,^39^,^40^. It can be shown that the synchronization problem for network systems with stochastic uncertainty can be written in this robust control form, where the loop gain directly translates to the synchronization margin. We refer the reader to supplementary material for more details and a mathematically rigorous discussion on the robust control-based interpretation behind the following mean square synchronization margin definition.

#### Mean Square Synchronization Margin

The equivalent Mean Square Synchronization Result is used to define the Mean Square Synchronization Margin as follows:
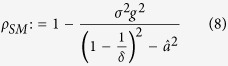
where 
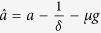
, 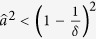, 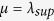, 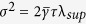, and 
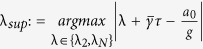
. Furthermore, 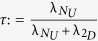, where 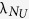 is the maximum eigenvalue of 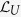 and 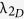 is the second-smallest eigenvalue of 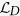.


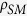
 measures the degree of robustness to stochastic perturbation. In particular, the larger the value of 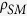 (i.e., the smaller the value of 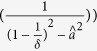, the larger the variance of stochastic uncertainty that can be tolerated in the network interactions before the network loses synchronization. When considering practical computation, it is important to emphasize 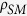, as computed by Eq. (8), is obtained from a sufficiency condition and hence is a guaranteed synchronization margin, i.e., the true synchronization margin will be larger than or equal to 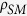. The synchronization condition for MSS of an *N*-node network system (2) as formulated in Eq. (8) is provided in terms of a scalar quantity instead of an *N*-dimensional matrix inequality. The condition is independent of network size, which makes it computationally attractive for large-scale networks. We now discuss the effects of various network parameters on synchronization.

#### Role of *τ* and 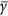


The parameter 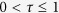 in 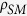 captures the effect of the uncertainty location in the graph topology. If the number of uncertain links 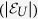 is large, the deterministic graph will become disconnected 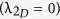, and thus *τ* will equal 1. In contrast, if a single link is uncertain 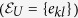, then 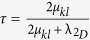. This indicates that the synchronization degradation is proportional to the link weight. Because 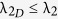, a lower algebraic connectivity of the deterministic graph further degrades 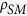. Thus, we can rank-order individual links within a graph with respect to their degradation of 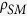, where a smaller *τ* produces an increased 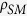. For example, it can be proved that the average value of *τ* for a nearest neighbour network is larger than that for a random network^8^. Thus, if a randomly chosen link is made stochastic in a nearest neighbour network and in a random network, the margin of synchronization decreases by a larger amount in the nearest neighbour network as compared than in the random network. We provide simulation results to support this claim in the supplementary information section. The significance of 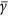 is straightforward, as it captures the maximum tolerable variance of the system, normalized with respect to the mean weight of the link. If 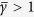, then the uncertainty occurring within the system is clustered, which leads to large intervals of high deviation. Similarly, if 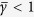, then the uncertainties are bundled closer to the mean value. Decreasing 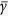 for the network increases 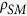.

#### Role of Laplacian Eigenvalues

The second smallest eigenvalue of the nominal graph Laplacian 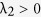 indicates the algebraic connectivity of the graph. Because 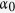 in (8) is a quadratic in λ, there exist critical values of 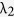 (or 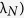 for a given set of system parameters and CoD below which (or above which) synchronization is not guaranteed. Hence, the critical 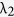 indicates that there is a required minimum degree of connectivity within the network for synchronization to occur. Furthermore, increasing the connectivity at appropriate nodes may increase 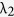, leading to higher 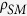. To understand the significance of 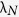, we look at the complement of the graph on the same set of nodes. We know from^41^ (Lemma provided in Supplementary Information for reference) that the sum of the largest Laplacian eigenvalue of a graph and the second smallest Laplacian eigenvalue of the complementary graph is a constant. Thus, if 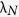 is large, then the complementary graph has low algebraic connectivity. Hence, a high 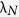 indicates the presence of many densely connected nodes. Therefore, we conclude that a robust synchronization is guaranteed for graphs with close-to-average node connectivity to graphs with isolated but highly connected hub nodes. Thus, decreasing 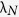 by reducing the connectivity of specific nodes (i.e., dense hub nodes) will help increase 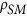.

#### Impact of Internal Dynamics

The internal dynamics are captured by parameters *a* and *δ*, which respectively represent the rate of linear instability and the bound on the rate of change of the nonlinearity. As *a* increases, the linear dynamics become more unstable. When all other parameters are held constant, an increase in *a* results in a decrease in 
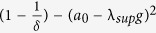
. Because 
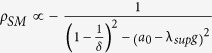
, an increase in *a* will produce a decrease in 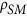. Thus, as the instability of the internal dynamics increases, the network becomes less robust to uncertainty. When the fluctuations in link weights are zero (i.e., CoD 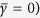, the critical value of 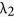 below which synchronization is not guaranteed is 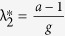. Furthermore, synchronization is not guaranteed for 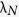 above the critical value 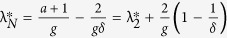. Thus, we see 
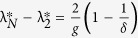
 and 
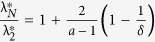
. While 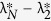 is independent of the internal dynamics parameter *a*, 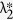 increases with an increase in *a*. In fact, for 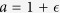, where 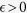 is arbitrarily small, we have 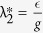. Hence, as the internal dynamics become more unstable, we require a higher degree of connectivity between the network agents to achieve synchronization. Because the nonlinearity 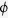 is sector-bounded by 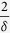, the impact of the nonlinearity on synchronization can be analysed using *δ*. When all of the other network parameters are held constant, 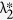 is independent of *δ* and 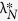 increases with increasing *δ*. Increasing the value of *δ* leads to an increase in 
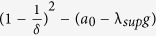
, which increases 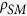. Hence, as the nonlinearity of the system is reduced, the system becomes more robust to uncertainties.

#### Impact of Coupling Gain

The impact of the coupling gain is more complicated than the impact of the internal dynamics. A very small coupling gain is not enough to guarantee 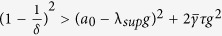, which is required to ensure 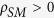. On the other hand, a very large coupling gain also does not guarantee 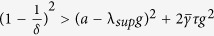. Thus, we can conclude the coupling gain affects the synchronization margin in a nonlinear fashion. Hence, to obtain the largest possible 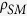, the network must operate at an optimal gain.

We now demonstrate how the main results of this paper can be used to determine the optimal value of the coupling gain 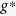 that maximizes the margin of synchronization for a given network topology (i.e., specific values of 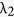 and 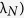 and uncertainty (i.e., CoD value 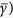. We assume that, for given values of 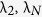, and 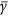, there exists a value of *g* for which synchronization is possible.

#### Optimal Gain

For the network system in Eq. (2) with 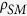 given by Eq. (8), the optimal gain 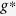 that produces the maximum 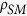 is
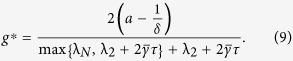


The derivation of this result will be discussed in the Methods Section. The results of the Mean Square Synchronization Margin 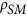 and the Optimal Gain 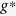 will be used in the following subsections to study the effect of neighbours and network connectivity on both nearest neighbour networks and random networks such as Erdos-Renyi and Small-World networks.

### Interplay of Internal Dynamics, Network Topology, and Uncertainty Characteristics

We now study the interplay of the internal dynamics (*a*), nonlinearity bound (*δ*), network topology (λ), and the uncertainty characteristics 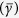 through simulations over a 1000-node network using a set of parameter values. To nullify the bias of uncertain link locations, we choose to work with a large number of uncertain links to obtain 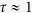.

**Figure 1:**
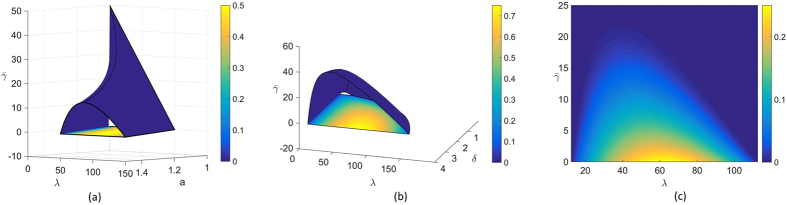
(**a**) 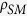 in 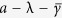 parameter space for 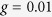 and 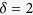, (**b**) 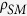 in 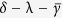 parameter space for 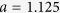 and 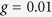, (**c**) 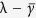 parameter space indicating 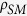 for 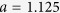, 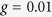, and 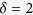.

In Fig. 1(a), we study the interplay of network topology, uncertainty, and the internal dynamics in the three-dimensional parameter space of 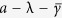. In Fig. 1(a), the region inside (or outside) the tunnel corresponds to the combination of parameter values where synchronization is possible (or not possible). Another important observation we make from Fig. 1(a) is that the area inside the tunnel increases with a decrease in either the internal instability or *a*. In Fig. 1(b), we plot the effects of changing the nonlinearity bound *δ* on the synchronization margin in the 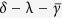 space. As *δ* is increased, the region of synchronization increases. Thus, a minimally nonlinear system is able to achieve synchronization even with high levels of communication. On the other hand, as the nonlinearity in a system becomes significant, the interaction between the nonlinearity and the fluctuations in the link weights could have adverse effects in a highly connected network. Intuitively, because a high communication amplifies the uncertainty between the agents, one might view this as the uncertainty in the fluctuations being wrapped around and amplified by the nonlinearity, which causes this high-communication desynchronization . In Fig. 1(c), we plot a slice of the synchronization regions from both Fig. 1(a,b) for 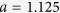, 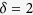, and 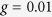, that highlights the synchronization margin.

### Optimal Neighbours in Nearest Neighbour Networks

**Figure 2:**
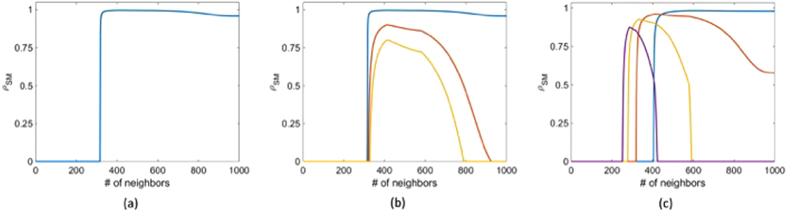
(**a**) Synchronization margin for 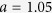, 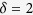, 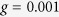, and 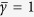 as the number of neighbours are varied in a nearest neighbour graph, (**b**) Synchronization margin for 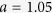, 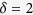, and 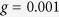 for different 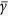 as the number of neighbours are varied in a nearest neighbour graph, where the blue, red, and yellow lines represent 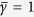, 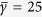, and 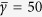, respectively, (**c**) Synchronization margin for 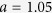, 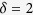, and 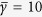 for different coupling gains as the number of neighbours are varied in a nearest neighbour graph, where the blue, red, yellow, and magenta lines indicate 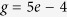, 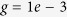, 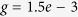, and 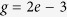, respectively.

The analytical formula for the synchronization margin in Eq. (8) provides us with a powerful tool to understand the effect of various network parameters on the synchronization margin. In this section, we investigate the effects of the number of neighbours on the synchronization margin. We consider a nearest neighbour network with 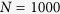 nodes and increase the number of neighbours to study their impact on the synchronization margin. The other network parameters are set to 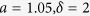, 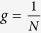, and 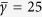. We choose a large number of uncertain links (70%) so that 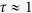 to remove the bias of uncertain link locations. We show the plot for the synchronization margin versus the number of neighbours in Fig. 2(a). From this plot, we see that there exists an optimal number of neighbours an agent requires in order to maximize the synchronization margin. Additionally, there is a minimum number of neighbours required by any given agent. Below this number, the network will not synchronize. However, an uncertain environment with too many neighbours is also detrimental to synchronization. This result highlights the fact that, while “good” information is propagated through neighbours via network interconnection, in an uncertain environment, these same neighbours can propagate “bad” information that is detrimental to reaching an agreement. In Fig. 2(b), we show the plot for the change in the synchronization margin versus a change in the number of neighbours for different values of CoD. For larger values of CoD, the drop in the margin as the network connectivity increases is more dramatic.

In light of the previous discussion, we can also interpret the coupling gain *g* as the amount of trust a given agent has in the information provided by its neighbours. In particular, if the coupling gain is large, then the agent has more trust in its neighbours. In Fig. 2(c), we show the effects of increasing the coupling gain on the synchronization margin. We observe that if an agent has more trust in its neighbours, then fewer neighbours are required to achieve synchronization. However, in an uncertain environment, an agent with more trust in its neighbours must avoid having more neighbours, as it is detrimental to synchronization. On the other hand, if an agent has less trust in its neighbours, more connections must be formed to gather as much information as possible, even if that information is corrupted. Thus, forging connections is good for a group with the goal of synchronization, but there exists a critical number of neighbours above which the benefits from forging new connections diminish.

### Optimal gain for complex networks

Based on the optimal gain formulation, we can now compare the performance of some well-known random networks and the optimal gain required to synchronize these networks. We use the following parameters in these simulations: the system instability 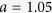, the nonlinearity bound 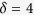, and the uncertainty statistics represented by CoD is 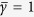. Furthermore, we choose 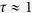. The properties of these random networks are studied for four different network sizes: 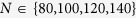, where *N* is the number of nodes.

**Figure 3:**
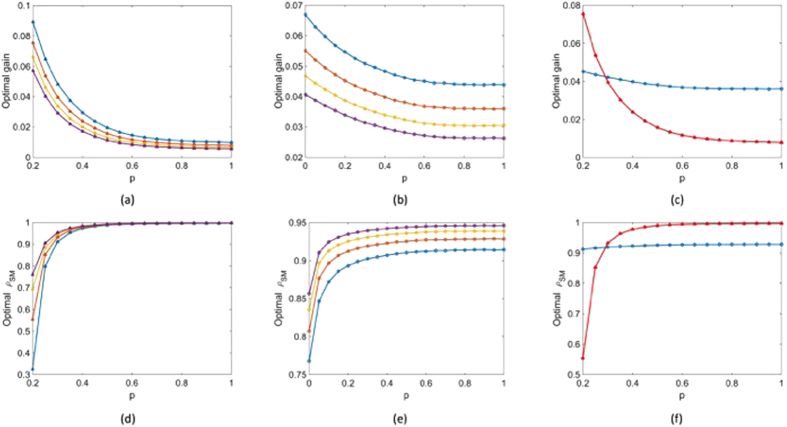
Optimal gain computation for (**a**) an Erdos-Renyi network with probability of connecting two nodes *p*, for varying network sizes and (**b**) a Small World network with probability of rewiring an edge *p*, for varying network sizes; (**c**) comparison of optimal gain for Erdos-Renyi and Small World networks as a function of probability for network size 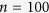. Optimal synchronization margin computation for (**d**) Erdos-Renyi network with probability of connecting two nodes *p*, for varying network sizes and (**e**) a Small World network with probability of rewiring an edge *p*, for varying network sizes; (**f**) comparison of optimal synchronization margin for Erdos-Renyi and Small World networks as a function of probability for network size 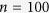. We provide the figure legends after the references. In (**a**,**b**,**d**,**e**), the blue, red, yellow, and magenta lines indicate *n* = 80, 100, 120, and 140, respectively. In (**c**,**f**), the blue and red lines indicate Small World and Erdos-Renyi networks respectively.

In Fig. 3(a), we plot the optimal gain for the Erdos-Renyi (ER) networks as a function of the edge connection probability. It is well known that for an Erdos-Renyi network of size *N* to be connected, the probability of connection must be 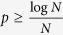. Hence, we plot these networks for probabilities ranging from 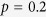 to 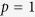. At 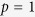, we obtain an all-to-all connection network, as each edge is connected with unit probability. In Fig. 3(d), we plot the corresponding optimal synchronization margin for the ER network. In Fig. 3(b,e), we plot the optimal gain and optimal synchronization margin, respectively, for a SW network with varying probability *p*^8^. To better observe the contrast in behaviour of both the ER and SW random networks, we plot in Fig. 3(c) the optimal gains for an ER network and an SW network with 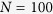 nodes.

We notice that, while a larger gain is required to synchronize the ER network than that for the SW network for smaller values of *p*, the optimal gain for the ER network is smaller than that of the SW network for larger values of *p*. In Fig. 3(d–f), we plot the optimal synchronization margins for the two networks. We notice an increase in the synchronization margin for the ER network around 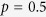. From these plots (specifically Fig. 3(c,f)), we conclude that for the given set of parameters, the ER (or SW) network has better synchronization properties (i.e., a smaller value of the optimal gain and a larger margin of synchronization) for larger (or smaller) values of *p*. The transition between the two cases occurs for some probability between 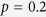 and 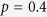.

## Discussion

We study the problem of synchronization in complex network systems in the presence of stochastic interaction uncertainty between the network nodes. We exploited the identical nature of the internal node dynamics to provide a sufficient condition for network synchronization. The unique feature of this sufficient condition is its independence from the network size. This makes the sufficient condition computationally attractive for large-scale network systems. Furthermore, this sufficient condition provides useful insight into the interplay between the internal dynamics of the network nodes, the network interconnection topology, the location of uncertainty, and the statistics of the uncertainty and into their effects on the network synchronization. The sufficient condition provided in the main result allows us to characterize the degree of robustness of a synchronized state to stochastic uncertainty through the definition of a mean square synchronization margin. Using the synchronization margin, a formulation for an optimal synchronization gain is derived to assist in designing gains for complex networks based purely on the system dynamics, nominal network Laplacian eigenvalues, and uncertainty statistics. This optimal gain result is used to compare various complex network topologies for given internal nodal dynamics.

When considered from a practical point of view, the synchronization margin is useful in determining the synchronizability of large-scale networks with stochastic uncertainty in the coupling. The independence of the result with respect to the network size can be used to obtain a bound on the tolerable uncertainty with minimal computational effort. In networked systems with communication uncertainty, these results can be used to provide a worst-case signal-to-noise ratio that is tolerable in communication or to design network connectivity in order to optimize the network’s tolerance to uncertainty. These results have potential applications in determining the optimal neighbours and coupling gain in consensus dynamics, swarm dynamics, and other situations where systems seek synchronization.

## Methods

### Mean Square Synchronization Condition

The system described by Eq. (2) is MSS as given by Definition 1, if there exist 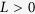, 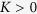 and 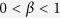, such that
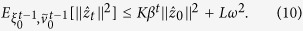


We refer to this as mean square stability of 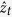. From Eq. (5), we obtain, 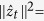

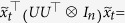
 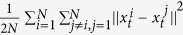, since 
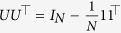
. Now, suppose there exist 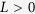, 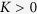, and 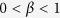, such that (10) holds true. We can rewrite (10) as
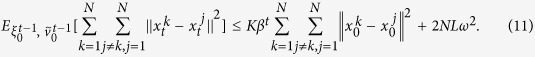


Thus, from (11) we obtain systems 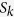 and 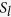, that satisfy (3) for mean square synchronization, where 
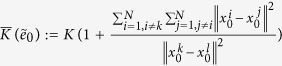
 and 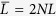.

In the Mean Square Synchronization Condition, we proved the mean square stability of (5) guarantees the MSS of (2). We will now utilize this result to provide a sufficiency condition for MSS of (5).

### Mean Square Stability of the Reduced System

The system given by (5) is mean square stable, if there exists a Lyapunov function 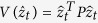 for a symmetric matrix 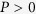, such that for some symmetric matrix 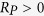 and 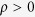 we have,
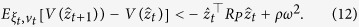


Consider 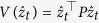 for a symmetrix matrix 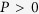, we know there exist 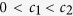, such that 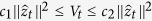. Let 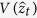 satisfy (12). Substituting 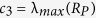 as the spectral radius of 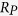 in (12) and using 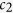 sufficiently large to define 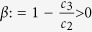, we obtain, 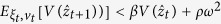. Taking expectation over 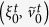 recursively, we obtain, 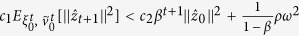. This guarantees the mean square stability of 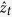, for 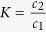 and 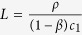.

We now utilize the Mean Square Stability of the Reduced System to define the Mean Square Synchronization Margin as given in (8). Towards this aim, we first construct an appropriate Lyapunov function, 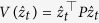, that guarantees mean square stability. From (5), defining 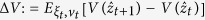, we obtain,
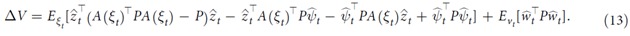


Now, suppose for some 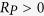, *P* satisfies,
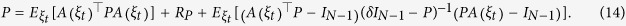


Using (14) and algebraic manipulations as given in^42^, we can rewrite 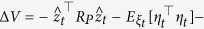 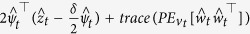, where 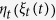 is given by 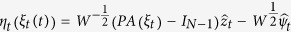 and 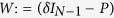. Since, 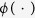 is monotonic and globally Lipschitz with constant 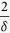, we know 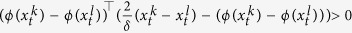. This gives 
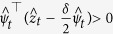
. Using this and writing 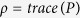, we obtain Eq. (12). Hence, (14) is sufficient for MSS of (1) from condition for Mean Square Stability of the Reduced System. Furthermore, the Eq. in (14) can be rewritten using^43^ (Proposition 12.1,1) as
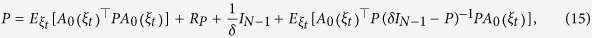
where 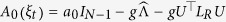 and 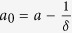. We observe this condition requires us to find a symmetric Lyapunov function matrix *P* of order 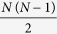. We now reduce the order of computation by using network properties. For this, consider 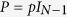, where 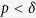 is a positive scalar. This gives us 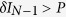. Using this and (5), we rewrite the condition in (15) as follows,
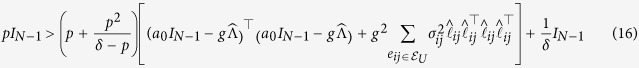


We know 
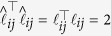
 and 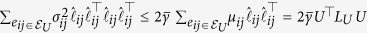. For 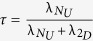, we have, 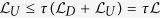. Hence, 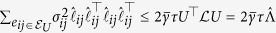. Substituting this into (16), a sufficient condition for inequality (16) to hold is given by 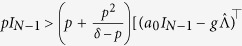

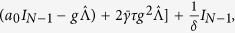
 a block diagonal equation. The individual blocks provide the sufficient condition for MSS as, 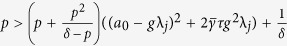, for all eigenvalues 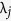 of 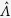. This is simplified as
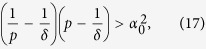
where *δ* > *p* > 0 and 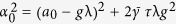 for all 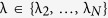 are eigenvalues of the nominal graph Laplacian. Now, for each of these conditions to hold true, we must satisfy condition (17) for the minimum value of 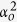 with respect to all possible λ. Now, λ^*^ that provides minimum values for 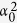 is found by setting 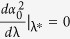, giving us 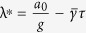. Using λ^*^, we know for (17) to be satisfied for all 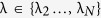, it must satisfy (17) for the farthest such λ from 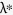. Since eigenvalues of the nominal graph Laplacian are positive and monotonic non-decreasing, all we need is to satisfy (17) for 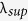, where 
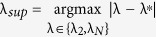
.

We observe from (17), if 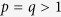 is a solution of (17), then 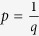. We state that (17) holds, if and only if,
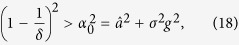
where 
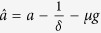
, 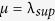, 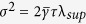. The “only if” part is obvious as, 
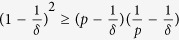
, from AM-GM inequality. To show the “if” part assume there exists 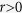, such that, 
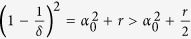
. Now consider some 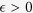 such that, 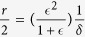. Hence, we obtain, 
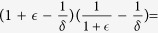

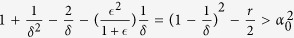
. Setting 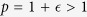, we know (17) holds true for some 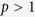. Hence, (17) and (18) are equivalent conditions. We now use (18) to define 
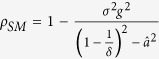
. The rationale for this and connections with existing conditions in robust control theory are discussed in the supplementary information.

We now provide the optimal coupling gain for systems with fixed internal dynamics interacting over a nominal network with a given set of uncertain links and 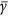. We observe from (18), to maximize the synchronization margin with respect to the coupling gain, *g*, we must minimize 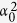, with respect to *g*, and maximize 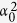, with respect to λ. This is a regular saddle-point optimization problem^44^. Hence, for a given λ, 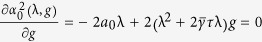. This provides us with the optimal gain as 
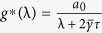
 with 
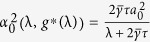
. The only important eigenvalues of the nominal graph Laplacian imposing limitations on synchronization, are 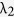 and 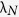. Hence we obtain 
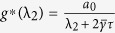
 and 
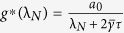
. Since 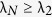, we have 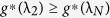, and 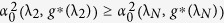.

There also exists a value of gain, 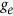, which provides the exact same synchronization margin for both 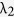 and 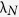. This is obtained by equating 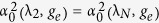, which provides, 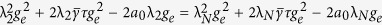. This gives us, for 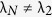, and 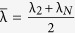, 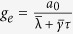. Furthermore, the 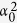 value for 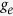, is given by, 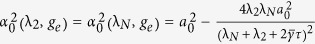. Since, 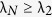, we have 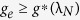. Furthermore, 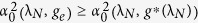, and, 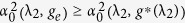. We also conclude that, 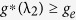, iff 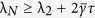 and 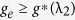, iff 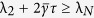. We observe that, 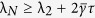, iff, 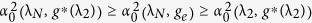. Hence, 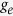, being the saddle-point solution, is the optimal gain providing the largest possible 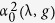, and the smallest 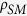. Similarly, 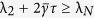, iff, 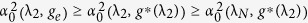. This gives 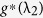 as the optimal gain. Furthermore, at the optimal gain, we always have 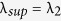. Defining, 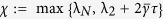, we can write the optimal gain, 
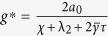
. Hence, for 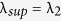, we obtain, 
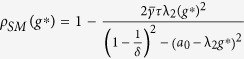
.

## Additional Information

**How to cite this article**: Diwadkar, A. and Vaidya, U. Limitations and tradeoffs in synchronization of large-scale networks with uncertain links. *Sci. Rep.* **6**, 21157; doi: 10.1038/srep21157 (2016).

## Supplementary Material

Supplementary Information
