# Supplementary material for: Limitations and tradeoffs in synchronization of large-scale networks with uncertain links
Source: Sci Rep. 2016 Apr 12;6:21157. doi: 10.1038/srep21157 (PMC4828643; doi:10.1038/srep21157)
Supplement: Supplementary Information [file srep21157-s1.pdf]

Limitations and tradeoffs in synchronization of large-scale networks with uncertain links

Amit Diwadkar and Umesh Vaidya
Electrical and Computer Engineering, Iowa State University
Coover Hall, Ames, IA, U.S.A. 50011

SUPPLEMENTARY INFORMATION

We consider the problem of synchronization in large-scale nonlinear network systems, where the scalar dynamics of the individual subsystem is assumed,

$$x_{t+1}^k = ax_t^k - \phi(x_t^k) + v_t^k \quad k = 1, \ldots, N, \tag{1}$$

where $x^k \in \mathbb{R}$ are the states of the kth subsystem, $a > 0$, and $v^k \in \mathbb{R}$ is an independent identically distributed (i.i.d.) additive noise process with zero mean (i.e., $E[v_t^k] = 0$) and variance $E[(v_t^k)^2] = \omega^2$. Subscript t denotes the index of the discrete time-step throughout the paper. The function, $\phi\colon \mathbb{R} \to \mathbb{R}$, is a monotonic, globally Lipschitz function with $\phi(0) = 0$ and Lipschitz constant, $\frac{2}{\delta}$. We assume the individual subsystems are linearly coupled over an undirected network given by a graph $G = (V, \mathcal{E})$ with node set V, edge set $\mathcal{E}$, and edge weights $\mu_{ij} \in \mathbb{R}^+$ for $i, j \in V$ and $e_{ij} \in \mathcal{E}$. Let $\mathcal{E}_U \subseteq \mathcal{E}$ be a set of uncertain edges and $\mathcal{E}_D = \mathcal{E} \setminus \mathcal{E}_U$. The weights for $e_{ij} \in \mathcal{E}_U$ are random variables: $\zeta_t^{ij} = \mu_{ij} + \xi_t^{ij}$, where μ_{ij} models the nominal edge weight and ξ_t^{ij} models the zero-mean uncertainty ($E[\xi_t^{ij}] = 0$), for all t, with variance $E[(\xi_t^{ij})^2] = E\left[(\zeta_{ij} - \mu_{ij})^2\right] = \sigma_{ij}^2$, for all t. If the coupling gain is $g > 0$, the individual agent dynamics of the coupled subsystem is given by,

$$x_{t+1}^k = ax_t^k - \phi\left(x_t^k\right) + g \sum_{e_{kj} \in \mathcal{E}_D} \mu_{kj}(x_t^j - x_t^k) + g \sum_{e_{kj} \in \mathcal{E}\mathcal{E}_U} (\mu_{kj} + \xi_t^{kj})(x_t^j - x_t^k) + v_t^k, \tag{2}$$

$$= ax_t^k - \phi\left(x_t^k\right) + g \sum_{e_{kj} \in \mathcal{E}} \mu_{kj}(x_t^j - x_t^k) + g \sum_{e_{kj} \in \mathcal{E}_U} \xi_t^{kj}(x_t^j - x_t^k) + v_t^k, \tag{3}$$

$$= ax_t^k - \phi\left(x_t^k\right) - g \left(\sum_{e_{kj} \in \mathcal{E}} \mu_{kj}\right) x_t^k + g \sum_{e_{kj} \in \mathcal{E}} \mu_{kj} x_t^j - g \left(\sum_{e_{kj} \in \mathcal{E}_U} \xi_t^{kj}\right) x_t^k + g \sum_{e_{kj} \in \mathcal{E}_U} \xi_t^{kj} x_t^j + v_t^k. \tag{4}$$

We denote the nominal graph Laplacian by $\mathcal{L} := [l(ij)] \in \mathbb{R}^{N \times N}$ and uncertain graph Laplacian by $\mathcal{L}_R := [l_R(ij)] \in \mathbb{R}^{N \times N}$, where

$$l(ij) = \begin{cases} -\mu_{ij}, & \text{if } i \neq j, \text{ and, } e_{ij} \in \mathcal{E} \\ \sum_{e_{ij} \in \mathcal{E}} \mu_{ij}, & \text{if } i = j \end{cases}, \quad \text{and,} \quad l_R(ij) = \begin{cases} -\xi_t^{ij}, & \text{if } i \neq j, \text{ and, } e_{ij} \in \mathcal{E}_U \\ \sum_{e_{ij} \in \mathcal{E}_U} \xi_t^{ij}, & \text{if } i = j \end{cases}. \tag{5}$$

The nominal graph Laplacian $\mathcal{L}$ is a sum of the graph Laplacian for the purely deterministic graph $(V, \mathcal{E}_D)$, and of the mean Laplacian for the purely uncertain graph $(V, \mathcal{E}_U)$. Hence, $\mathcal{L}$ may be written as $\mathcal{L} = \mathcal{L}_D + \mathcal{L}_U$, where $\mathcal{L}_D$, is the Laplacian for the graph over V with edge set $\mathcal{E}_D$. $\mathcal{L}_U$ is the mean Laplacian for the graph over V with edge set $\mathcal{E}_U$. We combine the individual systems to create the network system $(\tilde{x}_t)$ written as,

$$\tilde{x}_{t+1} = (aI_N - g(\mathcal{L} + \mathcal{L}_R))\, \tilde{x}_t - \tilde{\phi}\left(\tilde{x}_t\right) + \tilde{v}_t, \tag{6}$$

where I_N is the $N \times N$ identity matrix, $\tilde{x}_t = [x_t^1 \;\cdots\; x_t^N]^\top$, and $\tilde{\phi}(\tilde{x}_t) = [\phi_t^1(x_t^1) \;\cdots\; \phi_t^N(x_t^N)]^\top$.

Given the stochastic nature of the network system, we propose the following definition for mean square exponential synchronization [1].

Definition 1 (Mean Square Synchronization) *The network system* (6) *is said to be mean square synchronizing (MSS), if there exist positive constants,* $\beta < 1$, $\bar{K}(\tilde{e}_0) < \infty$, *and* $L < \infty$, *such that,*

$$E_{\xi_0^t, \tilde{v}_0^t} \parallel x_t^k - x_t^j \parallel^2 \leq \bar{K}(\tilde{e}_0)\beta^t \parallel x_0^k - x_0^j \parallel^2 + L\omega^2, \tag{7}$$

$\forall k, j \in [1, N]$, *where* $\tilde{e}_0$ *is a function of difference* $\parallel x_0^i - x_0^\ell \parallel^2$ *for* $i, \ell \in [1, N]$ *and* $\bar{K}(0) = K$ *for some constant* K.

Remark 2 *In the absence of additive noise,* $\tilde{v}_t$, *in system Eq.* (6), *the term* $L\omega^2$ *in Eq.* (7) *vanishes and Definition*

1 then reduces to mean square exponential (MSE) synchronization [2].

Mean Square Synchronization Result

Since the subsystems are identical, the synchronization manifold is spanned by the vector, $\mathbb{1} = [1, \ldots, 1]^\top$. The dynamics on the synchronization manifold are decoupled from the dynamics off the manifold and are essentially described by the dynamics of the individual system, which could be stable, oscillatory, or complex in nature. We now apply a change of coordinates to decompose the system dynamics on and off the synchronization manifold. Let $\mathcal{L} = V\Lambda V^\top$, where V is an orthonormal set of vectors given by $V = \left[\frac{\mathbb{1}}{\sqrt{N}} \;\; U\right]$, and U is a set of orthonormal vectors also orthonormal to $\mathbb{1}$. Furthermore, $\Lambda = \text{diag}\{\lambda_1, \cdots, \lambda_N\}$, where $0 = \lambda_1 < \lambda_2 \leq \cdots \leq \lambda_N$ are the eigenvalues of $\mathcal{L}$. Let $\tilde{z}_t = V^\top \tilde{x}_t$ and $\tilde{w}_t = V^\top \tilde{v}_t$. Multiplying (6) from the left side by $V^\top$, we obtain

$$\tilde{z}_{t+1} = \left(aI_N - g\left(V^\top(\mathcal{L} + \mathcal{L}_R)V\right)\right)\tilde{z}_t - \tilde{\psi}\left(\tilde{z}_t\right) + \tilde{w}_t, \tag{8}$$

where $\tilde{\psi}(\tilde{z}_t) = V^\top \tilde{\phi}\left(\tilde{x}_t\right)$. We can now write $\tilde{z}_t = \left[\begin{array}{cc} \bar{x}_t^\top & \hat{z}_t^\top \end{array}\right]^\top$, $\tilde{\psi}(\tilde{z}_t) := \left[\begin{array}{cc} \bar{\phi}_t^\top & \hat{\psi}_t^\top \end{array}\right]^\top$, and $\tilde{w}_t := \left[\begin{array}{cc} \bar{v}_t^\top & \hat{w}_t^\top \end{array}\right]^\top$, where

$$\bar{x}_t := \frac{\mathbb{1}^\top}{\sqrt{N}}\tilde{x}_t = \frac{1}{\sqrt{N}}\sum_{k=1}^{N} x_t^k, \quad \hat{z}_t := U^\top \tilde{x}_t \tag{9}$$

$$\bar{\phi}_t := \frac{\mathbb{1}^\top}{\sqrt{N}}\tilde{\phi}\left(\tilde{z}_t\right) = \frac{1}{\sqrt{N}}\sum_{k=1}^{N} \phi(x_t^k), \quad \hat{\psi}_t := U^\top \tilde{\phi}\left(\tilde{x}_t\right). \tag{10}$$

Furthermore, we have

$$E_{\tilde{v}}[\bar{v}_t^2] = \sqrt{N}\omega^2, \; E_{\tilde{v}}[\hat{w}_t\hat{w}_t^\top] = U^\top E_{\tilde{v}}[\tilde{v}_t\tilde{v}_t^\top]U = \omega^2 I_{N-1}. \tag{11}$$

From (8), we obtain

$$\begin{aligned} \bar{x}_{t+1} &= a\bar{x}_t - \bar{\phi}\left(\bar{x}_t\right) + \bar{v}_t \\ \hat{z}_{t+1} &= \left(aI_{N-1} - g\left(\hat{\Lambda} + U^\top \mathcal{L}_R U\right)\right)\hat{z}_t - \hat{\psi}_t + \hat{w}_t, \end{aligned} \tag{12}$$

where $\hat{\Lambda} = \text{diag}\{\lambda_2, \cdots, \lambda_N\}$. For the synchronization of system (6), we only need to demonstrate the mean square stability to the origin of the $\hat{z}$ dynamics, as given in (12). This feature is exploited to derive the sufficiency condition for mean square synchronization of the coupled system, as shown in the following lemma.

Lemma 3 *The system described by Eq. (6) is MSS, as given by Definition 1, if there exists $L > 0$, $K > 0$, and $0 < \beta < 1$, such that*

$$E_{\xi_0^t, \tilde{v}_0^t}\left[\| \hat{z}_t \|^2\right] \leq K\beta^t \| \hat{z}_0 \|^2 + L\omega^2. \tag{13}$$

Proof. To prove this result, we show the second moment of $\hat{z}_t$ dynamics is equivalent to the mean square error dynamics for each pair of systems. Then, we apply the stability results to the error dynamics to complete the proof. Consider Eq. (12). We have

$$\| \hat{z}_t \|^2 = \hat{z}_t^\top \hat{z}_t = \tilde{x}_t^\top \left(UU^\top \otimes I_n\right)\tilde{x}_t. \tag{14}$$

Then, we have $UU^\top = VV^\top - \frac{\mathbf{1}}{\sqrt{N}}\frac{\mathbf{1}^\top}{\sqrt{N}} = I_N - \frac{1}{N}\mathbf{1}\mathbf{1}^\top$. Substituting in (14), we obtain

$$\| \hat{z}_t \|^2 = \frac{1}{2N}\sum_{i=1}^{N}\sum_{j\neq i, j=1}^{N}\left(x_t^i - x_t^j\right)^\top\left(x_t^i - x_t^j\right). \tag{15}$$

Now, suppose there exist $L > 0$, $K > 0$, and $0 < \beta < 1$, such that

$$E_{\xi_0^t, \tilde{v}_0^t}\left[\| \hat{z}_t \|^2\right] \leq K\beta^t \| \hat{z}_0 \|^2 + L\omega^2. \tag{16}$$

This can be rewritten as

$$E_{\xi_0^t,\tilde{v}_0^t}\left[\sum_{k=1}^{N}\sum_{j\neq k,j=1}^{N} \parallel x_t^k - x_t^j \parallel^2\right] \leq K\beta^t \sum_{k=1}^{N}\sum_{j\neq k,j=1}^{N} \parallel x_0^k - x_0^j \parallel^2 + L\omega^2. \tag{17}$$

This implies

$$\sum_{k=1}^{N}\sum_{j\neq k,j=1}^{N} E_{\xi_0^t}\left[\parallel x_t^k - x_t^j \parallel^2\right] \leq K\beta^t \sum_{k=1}^{N}\sum_{j\neq k,j=1}^{N} \parallel x_0^k - x_0^j \parallel^2 + L\omega^2. \tag{18}$$

Thus, from (18) we obtain for all systems, S_k and S_l,

$$E_{\xi_0^t} \parallel x_t^k - x_t^l \parallel^2 \leq \bar{K}(\tilde{e}_0)\beta^t \parallel x_0^k - x_0^l \parallel^2 + L\omega^2, \tag{19}$$

where $\bar{K}(\tilde{e}_0) := K\left(1 + \frac{\sum_{i=1,i\neq k}^{N}\sum_{j=1,j\neq i}^{N}\|x_0^i - x_0^j\|^2}{\|x_0^k - x_0^l\|^2}\right)$. Hence, the proof. ■

We will now provide a slightly modified Eq. (12). We know $\mathcal{L}_R = \sum_{e_{ij}\in\mathcal{E}_U} \xi_t^{ij}\ell_{ij}\ell_{ij}^\top$, where $\ell_{ij} \in \mathbb{R}$ has values 1 and -1 in i^{th} and j^{th} entries, respectively, the remaining values are zeros. Thus, $\ell_{ij}^\top\ell_{ij} = 2$ for all $e_{ij} \in \mathcal{E}$. Hence, if $\hat{\ell}_{ij} = U^\top\ell_{ij}$, we calculate

$$U^\top\mathcal{L}_R U_d = \sum_{e_{ij}\in\mathcal{E}_U} \xi_t^{ij} U^\top \ell_{ij}\ell_{ij}^\top U = \sum_{e_{ij}\in\mathcal{E}_U} \xi_t^{ij}\hat{\ell}_{ij}\hat{\ell}_{ij}^\top, \tag{20}$$

where $\hat{\ell}_{ij}^\top\hat{\ell}_{ij} = 2$. Thus, we can write Eq. (12) as

$$\hat{z}_{t+1} = \left(aI_{N-1} - g\hat{\Lambda} - \sum_{e_{ij}\in\mathcal{E}_U} \xi_t^{ij}\hat{\ell}_{ij}\hat{\ell}_{ij}^\top\right)\hat{z}_t - \hat{\psi}(\hat{z}_t) + \hat{w}_t. \tag{21}$$

In Lemma 3, we prove mean square exponential stability of (21) guarantees the mean square synchronization of the coupled network of Lure systems, as given by (6). We will now utilize this to provide the sufficiency condition for mean square stabilization of Lure systems interacting over a network.

Theorem 4 *The network system in Eq.* (6) *is mean square synchronizing, if there exists a positive constant, p, that satisfies* $\delta > p$,

$$\left(p - \frac{1}{\delta}\right)\left(\frac{1}{p} - \frac{1}{\delta}\right) > \alpha_0^2, \tag{22}$$

where $\alpha_0^2 = (a_0 - \lambda_{sup}g)^2 + 2\bar{\gamma}\tau\lambda_{sup}g^2$, $a_0 = a - \frac{1}{\delta}$, *and* $\lambda_{sup} = \underset{\lambda\in\{\lambda_2,\lambda_N\}}{argmax}\left|\lambda + \bar{\gamma}\tau - \frac{a_0}{g}\right|$. *Furthermore,* $\tau := \frac{\lambda_{N_U}}{\lambda_{N_U}+\lambda_{2_D}}$, *where* λ_{N_U} *is the maximum eigenvalue of* $\mathcal{L}_U$, *and* λ_{2_D} *is the second smallest eigenvalue of* $\mathcal{L}_D$.

Proof. We first construct an appropriate Lyapunov function, $V(\hat{z}_t) = \hat{z}_t^\top P\hat{z}_t$, that guarantees mean square stability. From (21), defining $\Delta V := E_{\xi_t,v_t}\left[V(\hat{z}_{t+1}) - V(\hat{z}_t)\right]$, we obtain,

$$\Delta V = E_{\xi_t,\tilde{v}_t}\left[\hat{z}_t^\top\left(A(\xi_t)^\top PA(\xi_t) - P\right)\hat{z}_t - \hat{z}_t^\top A(\xi_t)^\top P\hat{\psi}_t - \hat{\psi}_t^\top PA(\xi_t)\hat{z}_t + \hat{\psi}_t^\top P\hat{\psi}_t\right] + E_{v_t}[\hat{w}_t^\top P\hat{w}_t]. \tag{23}$$

Now suppose for some $R_P > 0$, P satisfies

$$P = E_{\xi_t}\left[A(\xi_t)^\top PA(\xi_t)\right] + R_P + E_{\xi_t}\left[\left(A(\xi_t)^\top P - I_{N-1}\right)\left(\delta I_{N-1} - P\right)^{-1}\left(PA(\xi_t) - I_{N-1}\right)\right]. \tag{24}$$

Using (24) and algebraic manipulations as given in [3], we can rewrite ΔV as

$$\Delta V = -\hat{z}_t^\top R_P\hat{z}_t - E_{\xi_t}\left[\eta_t^\top\eta_t\right] - 2\hat{\psi}_t^\top\left(\hat{z}_t - \frac{\delta}{2}\hat{\psi}_t\right) + \text{trace}(PE_{v_t}[\hat{w}_t\hat{w}_t^\top]), \tag{25}$$

where $\eta_t(\xi_t(t))$ be given by $\eta_t(\xi_t(t)) = W^{-\frac{1}{2}} \left(PA(\xi_t) - I_{N-1}\right) \hat{z}_t - W^{\frac{1}{2}} \hat{\psi}_t$ and $W := (\delta I_{N-1} - P)$. Since $\phi(\cdot)$ is monotonic and globally Lipschitz with constant $\frac{2}{\delta}$, we know $\left(\phi\left(x_t^k\right) - \phi\left(x_t^l\right)\right)^\top \left(\frac{2}{\delta}\left(x_t^k - x_t^l\right) - \left(\phi\left(x_t^k\right) - \phi\left(x_t^l\right)\right)\right) > 0$. This gives

$$\hat{\psi}_t^\top \left(\hat{z}_t - \frac{\delta}{2}\hat{\psi}_t\right) = \tilde{\phi}\left(\tilde{x}_t\right)^\top UU^\top \left(\frac{2}{\delta}\tilde{x}_t - \tilde{\phi}\left(\tilde{x}_t\right)\right) = \tilde{\phi}\left(\tilde{x}_t\right)^\top \left(I - \frac{1}{N}\mathbb{1}\mathbb{1}^\top\right)\left(\frac{2}{\delta}\tilde{x}_t - \tilde{\phi}\left(\tilde{x}_t\right)\right) \tag{26}$$

$$= \frac{1}{2N}\sum_{k=1}^{N}\sum_{l=1, l\neq k}^{N} \left(\phi\left(x_t^k\right) - \phi\left(x_t^l\right)\right)^\top \left(\frac{2}{\delta}\left(x_t^k - x_t^l\right) - \left(\phi\left(x_t^k\right) - \phi\left(x_t^l\right)\right)\right) > 0. \tag{27}$$

Using (26) and writing $\rho = \text{trace}(P)$, we obtain,

$$E_{\xi_t, v_t}\left[V(\hat{z}_{t+1})) - V(\hat{z}_t)\right] < -\hat{z}_t^\top R_P \hat{z}_t + \rho\omega^2. \tag{28}$$

Hence, (24) is sufficient for MSS of (1) from condition for Mean Square Stability of the Reduced System. Furthermore, the equation in (24) can be rewritten using [4] (Proposition 12.1,1) as

$$P = E_{\xi_t}\left[A_0(\xi_t)^\top P A_0(\xi_t)\right] + R_P + \frac{1}{\delta}I_{N-1} + E_{\xi_t}\left[A_0(\xi_t)^\top P(\delta I_{N-1} - P)^{-1} P A_0(\xi_t)\right], \tag{29}$$

where $A_0(\xi_t) = a_0 I_{N-1} - g\hat{\Lambda} - gU^\top \mathcal{L}_R U$ and $a_0 = a - \frac{1}{\delta}$. We observe this condition requires us to find a symmetric Lyapunov function matrix, P, of order $\frac{N(N-1)}{2}$. We now reduce the order of computation by using network properties. For this, consider $P = pI_{N-1}$, where $p < \delta$ is a positive scalar. This gives us $\delta I_{N-1} > P$. Using this and (21), we rewrite the condition in (29) as follows

$$\begin{aligned} pI_{N-1} > {} & p(a_0 I_{N-1} - g\hat{\Lambda})^\top (a_0 I_{N-1} - g\hat{\Lambda}) + \frac{p^2}{\delta - p}(a_0 I_{N-1} - g\hat{\Lambda})^\top (a_0 I_{N-1} - g\hat{\Lambda}) + \frac{1}{\delta}I_{N-1} \\ & + pg^2 \sum_{e_{ij}\in\mathcal{E}_U} \sigma_{ij}^2 \hat{\ell}_{ij}\hat{\ell}_{ij}^\top \hat{\ell}_{ij}\hat{\ell}_{ij}^\top + \frac{p^2 g^2}{\delta - p}\sum_{e_{ij}\in\mathcal{E}_U} \sigma_{ij}^2 \hat{\ell}_{ij}\hat{\ell}_{ij}^\top \hat{\ell}_{ij}\hat{\ell}_{ij}^\top. \end{aligned} \tag{30}$$

We know $\hat{\ell}_{ij}^\top \hat{\ell}_{ij} = \ell_{ij}^\top U_d U_d^\top \ell_{ij} = \ell_{ij}^\top \ell_{ij} = 2$ and

$$\sum_{e_{ij}\in\mathcal{E}_U} \sigma_{ij}^2 \hat{\ell}_{ij}\hat{\ell}_{ij}^\top \hat{\ell}_{ij}\hat{\ell}_{ij}^\top \leq 2\bar{\gamma}\sum_{e_{ij}\in\mathcal{E}_U} \mu_{ij}\hat{\ell}_{ij}\hat{\ell}_{ij}^\top = 2\bar{\gamma}U^\top \mathcal{L}_U U. \tag{31}$$

Now, suppose $\mathcal{L}_U \leq \tau\mathcal{L} = \tau\left(\mathcal{L}_D + \mathcal{L}_U\right)$. We then obtain $\tau \geq \frac{\lambda_{N_U}}{\lambda_{N_U} + \lambda_{2_D}}$. We choose τ as

$$\tau = \frac{\lambda_{N_U}}{\lambda_{N_U} + \lambda_{2_D}}. \tag{32}$$

Hence, we have $\mathcal{L}_U \leq \tau\left(\mathcal{L}_D + \mathcal{L}_U\right) = \tau\mathcal{L}$. Then, bounding $\mathcal{L}_U$ in (31) using (32), we obtain,

$$\sum_{e_{ij}\in\mathcal{E}_D} \sigma_{ij}^2 \hat{\ell}_{ij}\hat{\ell}_{ij}^\top \hat{\ell}_{ij}\hat{\ell}_{ij}^\top \leq 2\bar{\gamma}\tau U^\top \mathcal{L} U = 2\bar{\gamma}\tau\hat{\Lambda}. \tag{33}$$

Substituting (33) into (30), a sufficient condition for inequality (30) to hold is given by

$$pI_{N-1} > \left(p + \frac{p^2}{\delta - p}\right)(a_0 I_{N-1} - g\hat{\Lambda})^\top (a_0 I_{N-1} - g\hat{\Lambda}) + \left(p + \frac{p^2}{\delta - p}\right) 2\bar{\gamma}\tau g^2 \hat{\Lambda} + \frac{1}{\delta}I_{N-1}. \tag{34}$$

Equation (34) is a diagonal matrix equation that provides a sufficient condition for MSS as

$$p > \left(p + \frac{p^2}{\delta - p}\right)\left((a_0 - g\lambda_j)^2 + 2\bar{\gamma}\tau g^2\lambda_j\right) + \frac{1}{\delta}, \tag{35}$$

for all eigenvalues λ_j of $\hat{\Lambda}$. This is simplified as

$$\left(p - \frac{1}{\delta}\right)\left(\frac{1}{p} - \frac{1}{\delta}\right) > \alpha_0^2, \tag{36}$$

where $\delta > p > 0$ and $\alpha_0^2 = (a_0 - g\lambda)^2 + 2\bar{\gamma}\tau\lambda g^2$ for all $\lambda \in \{\lambda_2, \ldots, \lambda_N\}$ are eigenvalues of the nominal graph Laplacian. Now, for each of these conditions to hold true, we must satisfy condition (36) for the minimum value of α_o^2 with respect to all possible λ. Now, λ^* provides minimum values for α_0^2 found by setting $\left.\frac{d\alpha_0^2}{d\lambda}\right|_{\lambda^*} = 0$, giving us

$$\lambda^* = \frac{a_0}{g} - \bar{\gamma}\tau. \tag{37}$$

Using λ^*, we conclude, if (36) is to be satisfied for all $\lambda \in \{\lambda_2 \ldots, \lambda_N\}$, it must satisfy (36) for the farthest such λ from λ^*. Since eigenvalues of a graph Laplacian are positive and monotonic non-decreasing, all we need is to satisfy (36) for λ_{sup}, where $\lambda_{sup} = \underset{\lambda \in \{\lambda_2, \lambda_N\}}{\text{argmax}} |\lambda - \lambda^*|$. ■

In the following discussion we will provide a system theoretic interpretation to the proposed definition of mean square synchronization margin. For completion, we restate the definition for mean square synchronization margin.

Definition 5 (Mean Square Synchronization Margin) *The margin for synchronization for network system* (6) *is defined as*

$$\rho_{SM} := 1 - \frac{\sigma^2 g^2}{\left(1 - \frac{1}{\delta}\right)^2 - \hat{a}^2}, \tag{38}$$

where $\hat{a} = a - \frac{1}{\delta} - \lambda_{sup} g$, $\hat{a}^2 < \left(1 - \frac{1}{\delta}\right)^2$, *and* $\lambda_{sup} := \underset{\lambda \in \{\lambda_2, \lambda_N\}}{argmax} \left|\lambda + \bar{\gamma}\tau - \frac{a_0}{g}\right|$. *Furthermore,* $\tau := \frac{\lambda_{N_U}}{\lambda_{N_U} + \lambda_{2_D}}$, *where* λ_{N_U} *is the maximum eigenvalue of* $\mathcal{L}_U$ *and* λ_{2_D} *is the second smallest eigenvalue of* $\mathcal{L}_D$.

System Theoretic Interpretation of Syncronization Condition and Margin

The first step towards the system theoretic interpretation to margin definition is to show the stability condition derived in Theorem 4, i.e., Eq. (22), has the following equivalent,

$$\left(p - \frac{1}{\delta}\right)\left(\frac{1}{p} - \frac{1}{\delta}\right) > \alpha_0^2 \iff \left(1 - \frac{1}{\delta}\right)^2 > E_{\xi_t}[(\hat{a} - \xi_t g)^2]. \tag{39}$$

The main point of the equivalence is the equivalent stability condition on the right-hand side is independent of p. We observe from (36), if $p = q$ is a valid solution, so is $p = \frac{1}{q}$. Applying the inequality of arithematic and geometric mean (AM-GM Inequality) to p and $\frac{1}{p}$, from Eq. (36) we obtain,

$$\left(1 - \frac{1}{\delta}\right)^2 = 1 - \frac{2}{\delta} + \frac{1}{\delta^2} \geq 1 - \left(p + \frac{1}{p}\right)\frac{1}{\delta} + \frac{1}{\delta^2} > \alpha_0^2. \tag{40}$$

We know, if $p = q > 1$ is a solution of (48), then $p = \frac{1}{q}$ and $p = 1$ are also solutions, since we obtain from (40),

$$\left(1 - \frac{1}{\delta}\right)^2 > \left(p - \frac{1}{\delta}\right)\left(\frac{1}{p} - \frac{1}{\delta}\right) > E_{\xi_t}[(\hat{a} - \xi_t g)^2]. \tag{41}$$

Hence, we have for some $r > 0$,

$$\left(1 - \frac{1}{\delta}\right)^2 = E_{\xi_t}[(\hat{a} - \xi_t g)^2] + r > E_{\xi_t}[(\hat{a} - \xi_t g)^2] + \frac{r}{2}. \tag{42}$$

Now, consider some $\epsilon > 0$, such that

$$\frac{r}{2} = \left(\frac{\epsilon^2}{1 + \epsilon}\right)\frac{1}{\delta} = \left(1 + \epsilon + \frac{1}{1 + \epsilon} - 2\right)\frac{1}{\delta}. \tag{43}$$

From (43), we obtain,

$$\left(1+\epsilon-\frac{1}{\delta}\right)\left(\frac{1}{1+\epsilon}-\frac{1}{\delta}\right)=1+\frac{1}{\delta^2}-\frac{2}{\delta}-\left(1+\epsilon+\frac{1}{1+\epsilon}-2\right)\frac{1}{\delta}=\left(1-\frac{1}{\delta}\right)^2-\frac{r}{2}. \tag{44}$$

Using (42), and (44), and setting $p=1+\epsilon>1$, we obtain

$$\left(p-\frac{1}{\delta}\right)\left(\frac{1}{p}-\frac{1}{\delta}\right)=\left(1-\frac{1}{\delta}\right)^2-\frac{r}{2}>E_{\xi_t}\left[(\hat{a}-\xi_t g)^2\right]. \tag{45}$$

Hence, (40) is a necessary and sufficient condition for (22), implying equivalence (39).

One observes the sufficient condition, as provided in Theorem 4 (i.e., Eq. (22)), is a Riccati equation in one dimension. For the scalar i.i.d. random variable, ζ, writing $E[\zeta]=\mu:=\lambda_{sup}$, $E[(\zeta-\mu)^2]=\sigma^2:=2\bar{\gamma}\tau\lambda_{sup}$, we can write Eq. (22) as

$$p>E_\zeta\left[(a_0-\zeta g)^2 p+(a_0-\zeta g)^2\frac{p^2}{\delta-p}+\frac{1}{\delta}\right]. \tag{46}$$

In fact, the above condition is a sufficient condition for stability of the following scalar nonlinear system.

$$x_{t+1}=(a_0-\zeta g)\,x_t+\phi_0\,(x_t)=(a_0-\mu g)\,x_t+\phi_0\,(x_t)-\xi_t g x_t, \qquad \frac{1}{\delta}>\|\phi_0(x)\|_\infty, \tag{47}$$

where $\|\phi_0(\cdot)\|_\infty$ is the H_∞ of ϕ_0 ([6]) and $\xi_t=\zeta-\mu$ is a zero mean random variable with variance σ^2. The CoD for ζ_c is $\gamma_c=\frac{\sigma_c^2}{\mu_c}=2\bar{\gamma}\tau$. Equation (46) can be rewritten as

$$\left(p-\frac{1}{\delta}\right)\left(\frac{1}{p}-\frac{1}{\delta}\right)>E_{\zeta_c}\left[(a_0-\zeta_c g)^2\right]=(a_0-\mu_c g)^2+\sigma_c^2 g^2. \tag{48}$$

We would like to relate the above stability condition to results from robust stability theory. The central premise of this theory is, if the product of the two gains consisting of a system in the forward loop and the feedback loop is less than unity, then the feedback interconnection is stable. The product of the two gains is referred to as loop gain. In Fig. 1(a), we represent the dynamics of a scalar system (47) as a feedback interconnection of a mean linear system with two feedback loops consisting of nonlinearity and stochastic uncertainty. In the following, we analyze each of these feedback loops separately (as shown in Figs. 1(b) and 1(c)) to derive the stability condition in terms of loop gain. Then, we combine the two separate stability conditions to show the main result of this paper can alternatively be interpreted in terms of loop gains, thereby leading to the proposed definition of a synchronization margin.

Robust Stability to Norm-bounded Nonlinearity: In Fig. 1(b), we have a scalar linear system in the forward loop and a norm bounded nonlinearity in the feedback loop. The scalar system is given by

$$G: \quad x_{t+1}=\hat{a}x_t:=(a_0-\mu g)x_t, \tag{49}$$

where $a_0=a-\frac{1}{\delta}$ is the dynamics of the linear system with mean uncertainty in feedback and G represents the system with the transfer function, $G(z)=\frac{1}{z-\hat{a}}$. In our context, the feedback loop with gain, g, comes from the nominal network, but is not shown in the figure for simplicity of explanation. Furthermore, the linear dynamics is assumed stable. Results from robust stability and Small Gain Theorem [5, 6] can be used to study the stability of the closed loop. If $\|G\|_\infty$ represents the H_∞ norm of the system and $\|\phi_0\|_\infty$ denotes the H_∞ norm of the feedback nonlinearity, the closed loop is stable if

$$1>\|G\|_\infty\|\phi_0\|_\infty=\left(\frac{1}{1-|\hat{a}|}\right)\frac{1}{\delta}, \qquad \text{where} \qquad \|G\|_\infty=\frac{1}{1-|\hat{a}|}, \quad \|\phi_0\|_\infty=\frac{1}{\delta}. \tag{50}$$

The condition for robust stability provided in (50) can be reformulated as

$$\left(1-\frac{1}{\delta}\right)^2>\hat{a}^2. \tag{51}$$

Robust Stability to Stochastic Uncertainty: Using results developed in [7] under a setting of a linear time invariant system with vector states, we can analyze the stability of the feedback interconnection of a scalar linear system with stochastic uncertainty (as shown in Fig. (1)c). The condition for mean square stability of the closed loop

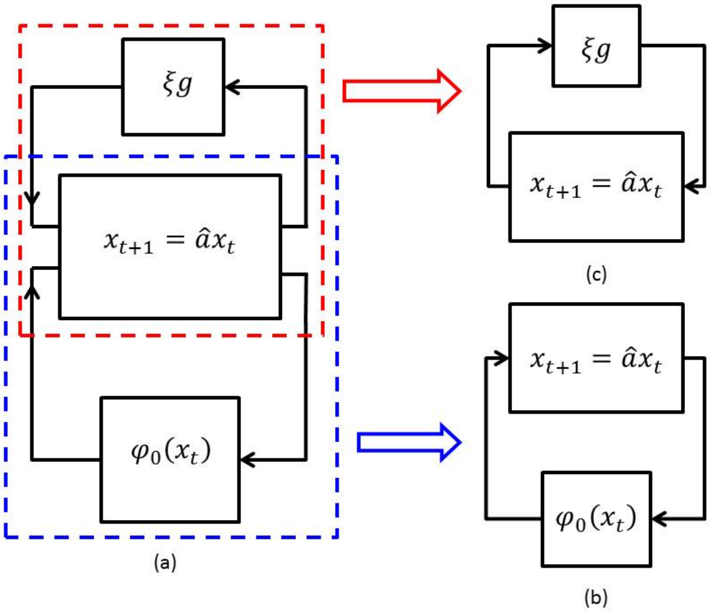

FIG. 1: (a) System with H_∞-norm bounded nonlinearity and stochastic uncertainty in feedback, (b) system with H_∞-norm bounded nonlinearity in feedback, and (c) system with stochastic uncertainty in feedback.

system, as obtained from the Small Gain Theorem in [7], is

$$1 > \|G\|_{MS}\|\xi_t g\|_{MS}, \tag{52}$$

where $\|G\|_{MS}$ and $\|\xi_t g\|_{MS}$ denote the mean square norm of the system and the stochastic uncertainty, respectively. It is shown in [7], that $\|G\|_{MS} = \|G\|_2^2$ for a scalar system, where $\|G\|_2$ denotes the H_2-norm of the transfer function $G(z)$. The H_2-norm of the scalar system $G(z)$ with an impulse response $h(k) = \hat{a}^k$ is given by

$$\|G\|_2 = \left(\sum_{k=0}^{\infty} h(k)^2\right)^{\frac{1}{2}} = \left(\sum_{k=0}^{\infty} \left(\hat{a}^k\right)^2\right)^{\frac{1}{2}}. \tag{53}$$

Thus, the mean square norm of the the linear system is given by

$$\|G\|_{MS}^2 = \sum_{k=0}^{\infty} \left(\hat{a}^2\right)^k = \frac{1}{1-\hat{a}^2}. \tag{54}$$

The mean square norm of the stochastic uncertainty with zero mean is simply given by its variance, i.e., $\|\xi_t g\|_{MS}^2 = \sigma^2 g^2$. The stability condition (52) for mean square stability of the feedback loop for the system in Fig. (1)c can now be formulated as

$$1 > \left(\frac{1}{1-\hat{a}^2}\right)\sigma^2 g^2 \quad \Longleftrightarrow \quad 1 > \hat{a}^2 + \sigma^2 g^2 = E_{\xi_t}\left[(\hat{a} - \xi_t g)^2\right]. \tag{55}$$

Robust Stability to Norm-bounded Nonlinearity and Stochastic Uncertainty: Stability conditions from the two individual feedback loops as discussed in the previous two sections can be combined to obtained the stability condition in terms of loop gain for the entire system as shown in Fig. 1(a). In particular, the condition for the mean square synchronization of the network, where both the norm-bounded nonlinearity and the stochastic uncertainty are present, can be written as

$$\left(1 - \frac{1}{\delta}\right)^2 > \hat{a}^2 + \sigma^2 g^2. \tag{56}$$

The stability condition in Eq. (56) is the correct method for combining the stability condition for the two individual feedback loops as expressed in Eqs. (51) and (55) for the following reasons. Using $E_{\xi_t}[(\hat{a}-\xi_t g)^2] = \hat{a}^2 + \sigma^2 g^2$ and the equivalent relationship (39), we notice (56) is exactly the mean square synchronization condition as derived in the main results of the paper. Furthermore, when $\sigma = 0$ (i.e., no stochastic uncertainty), condition (56) reduces to condition (51). Similarly, when $\delta = \infty$ (i.e., zero nonlinearity), then condition (56) reduces to (55). Hence, Eq. (56) can be viewed as the proper generalization of the stability conditions from the two individual loops to the entire system with these two loops operating in tandem.

Discussion on Synchronization Margin: Mean square synchronization condition as expressed in Eq. (56) can be used to derive the expression for mean square synchronization margin. In particular, we note condition (56), after algebraic manipulation, can equivalently be written as

$$\left(1-\frac{1}{\delta}\right)^2 > \hat{a}^2 + \sigma^2 g^2 \quad \iff \quad 1 > \left(\frac{1}{\left(1-\frac{1}{\delta}\right)^2 - \hat{a}^2}\right)\sigma^2 g^2. \tag{57}$$

The equivalent condition (57) has a nice system theoretic-based interpretation in terms of loop gain. In particular, the quantity, $\left(\frac{1}{\left(1-\frac{1}{\delta}\right)^2 - \hat{a}^2}\right)$, can be thought of as the gain of the mean linear system with nonlinearity in the feedback loop and $g^2\sigma^2$ as the gain of the stochastic uncertainty. The system will have a larger margin of stability, if the product of these two quantities is further from one. In particular, the smaller the quantity $\left(\frac{1}{\left(1-\frac{1}{\delta}\right)^2 - \hat{a}^2}\right)$, the greater the variance, $\sigma^2 g^2$, that can be tolerated to maintain stability, and, hence, more robust the system is to stochastic uncertainty and vice versa. This motivates us to propose the following definition of synchronization margin as a quantity, which measures how far the loop gain is from one.

$$\rho_{SM} = 1 - \left(\frac{1}{\left(1-\frac{1}{\delta}\right)^2 - \hat{a}^2}\right)\sigma^2 g^2. \tag{58}$$

This is precisely the definition of synchronization margin as proposed in Definition 5.

Interplay of Internal Dynamics, Network Topology and Uncertainty Characteristics

We now study the interplay of the internal dynamics (a), nonlinearity bound (δ), network topology (λ), and the uncertainty characteristics ($\bar{\gamma}$) through simulations, using a set of parameter values. To nullify the bias of uncertain link locations, we choose to work with a large number of uncertain links to obtain $\tau \approx 1$. In Fig. 2, we provide different orientations of the 3-dimensional plots used to discuss the interplay between various system, network, and uncertainty parameters over a network with 1000 nodes. In Figs. 2(a) and 2(b), we plot the boundary for the region with a positive ρ_{SM} in the $a-\lambda-\bar{\gamma}$ space, for $g = 0.01$, and $\delta = 2$. In Figs. 2(c) and 2(d), we plot the boundary for the region with a positive ρ_{SM} in the $a-\lambda-\bar{\gamma}$ space, for $g = 0.01$, and $a = 1.125$.

Location of Uncertainty and Network Topology

In the main document, we briefly discussed the impact of the parameter, τ, on the synchronization margin, ρ_{SM}. There exists an inverse relation between τ and ρ_{SM}, given by $\rho_{SM} = 1 - \tau\left(\frac{2\lambda_{sup}\bar{\gamma}g^2}{\left(1-\frac{1}{\delta}\right)^2 - \left(a-\frac{1}{\delta}-\lambda_{sup}g\right)^2}\right)$, thus, making higher τ detrimental for robustness (low value of ρ_{SM}). In this section, we will study the interplay between network topology and the location of uncertainty within the network. In particular, we wish to analyze the average robustness of networks to any single interconnection being uncertain. The idea is to observe if certain connectivity patterns make a network more robust to uncertainty at a single interconnection, averaged over all possible interconnections being individually uncertain. These ideas are aimed towards understanding the average impact of localized cyber-attacks on networks. To put things in perspective, a simple spatially invariant network will have the same τ value for any particular link being uncertain as the network structure is identical when any of the links are removed. However, if some of the links were to be changed to form different interconnections while keeping the number of edges the same, it might be possible to increase the robustness of the network. To ensure that we isolate the impact of location in this study, we assume unit link weights for all network links. For this analysis, we choose our networks to have emerged from a Small World phenomenon, a class of networks that emerge naturally in physical and social scenarios.

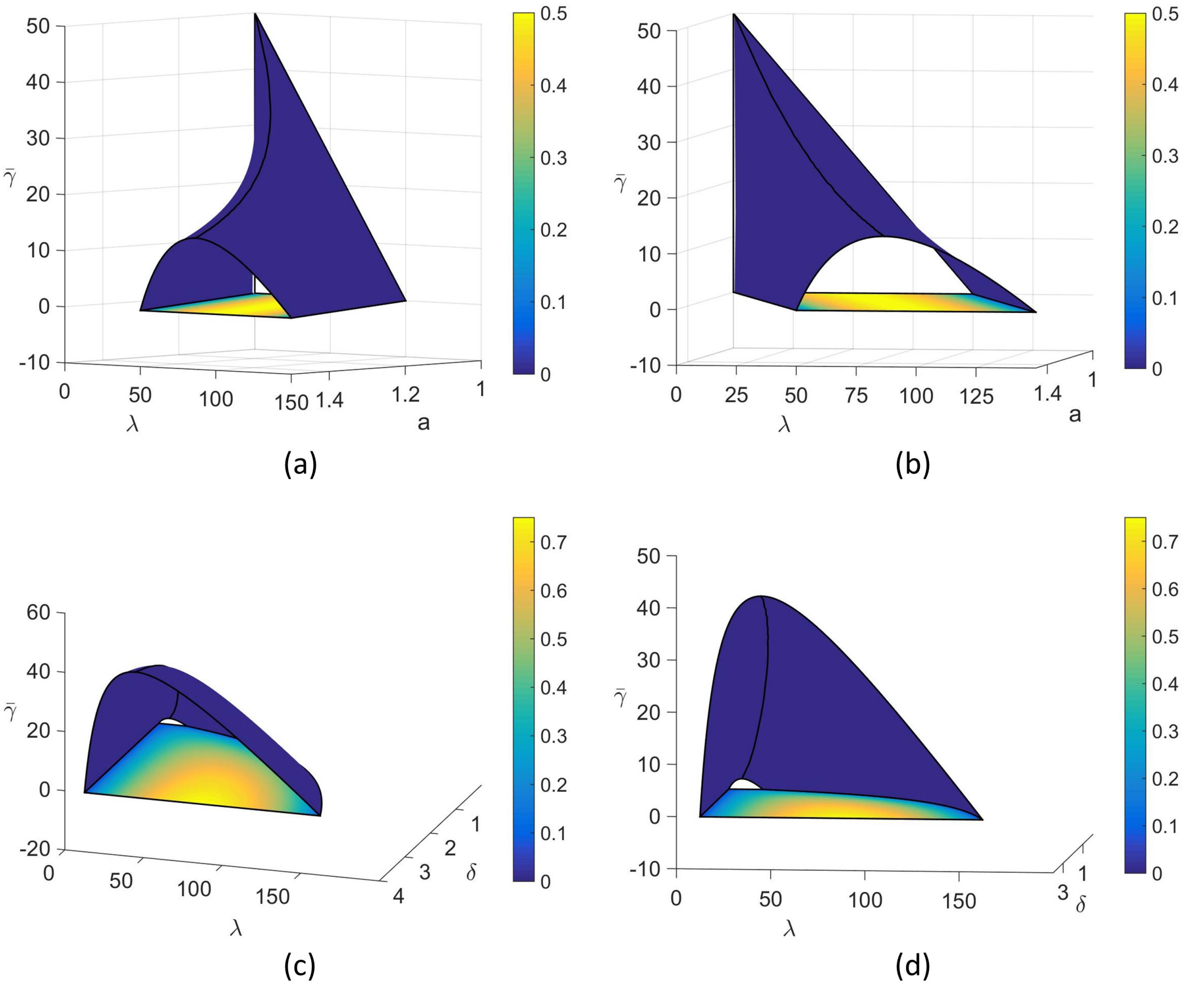

FIG. 2: (a)&(b) $a - \lambda - \bar{\gamma}$ parameter space indicating ρ_{SM} for $g = 0.01$, and $\delta = 2$, (c)&(d) $\delta - \lambda - \bar{\gamma}$ parameter space indicating ρ_{SM} for $a = 1.125$ and $g = 0.01$.

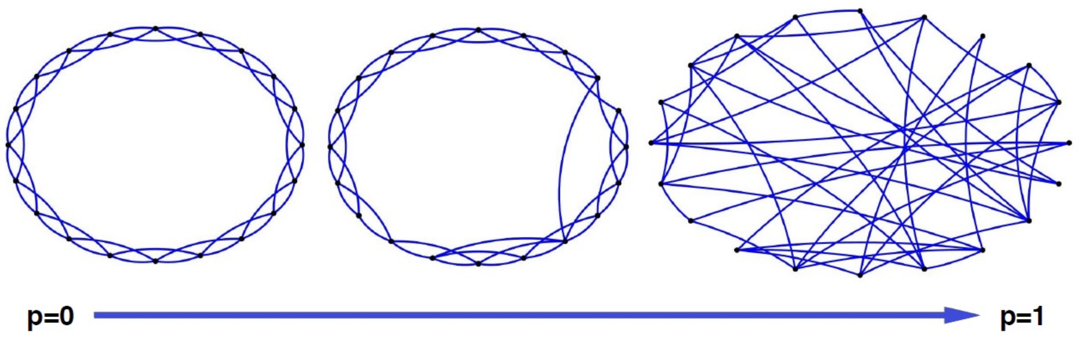

FIG. 3: Small World network connectivity graph with increasing value of rewiring probability p.

Small World networks, classified as *random networks*, [13] were first introduced in [8], and constructed from nearest neighbor networks with random rewiring of links between nodes with a chosen probability. The fundamental idea of the Small World phenomenon suggested that as networks emerge in a natural setting, even though most nodes connect

with neighbouring nodes, there is a chance of long distance interaction between nodes. The propensity of such long distance interactions is measured by the *rewiring probability* e. When the rewiring probability, $e = 0$, the network is a nearest neighbor network. As e increases, the network loses its nearest neighbor property and has an increasing number of long distance interconnections. This can be visualized through the schematic in Fig. 3, which shows the change in the network connectivity as the rewiring probability increases from $e = 0$ to $e = 1$ [8]. For the purpose of this study, we wish to understand if a higher proclivity for long distance interconnections is beneficial for emerging networks, especially when after a network has emerged, any particular interconnection could be subject to uncertainty in the form of attacks or fluctuations in the link weight. We would like to clarify that the rewiring probability of a Small World network has no relation to the interconnection uncertainty, and the link uncertainty is only studied for a fixed network, that has emerged through the Small World phenomenon for a given rewiring probability e. The procedure for this study is outlined in the following paragraph.

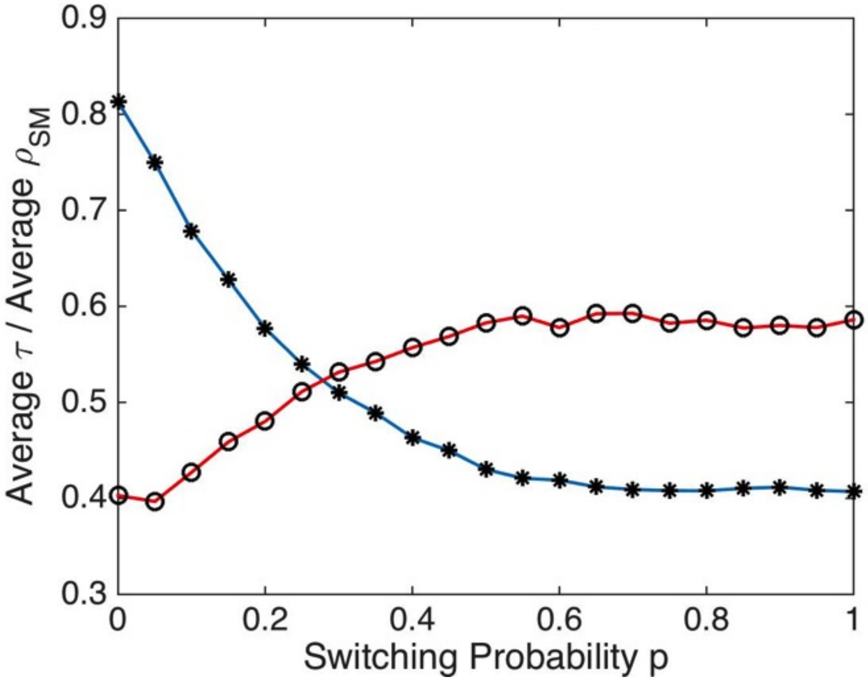

FIG. 4: Small World network average $\bar{\tau}_{avg}$ as a function of rewiring probability p. Figure legend is provided after the references. The blue line with a solid dot indicates the value of average τ, and the red line with the circle indicates the value of average ρ_{SM}, at the particular switching probability p

We initially consider a network of 50 nodes with 8 nearest neighbors per node. Then, we increase the rewiring probability from $p = 0$ to $p = 1$ in steps of 0.1 to obtain various random networks. For each such network, we cycle through all the individual links making them uncertain. Then, we compute the value of τ corresponding to the particular uncertain link with a unit mean value for the interconnection weight. These values of τ are used to find the average value of τ_{avg} for the given network. Since these networks are random in nature and the interconnections are formed by probabilistically rewiring links from a nearest neighbor network, we obtain the τ_{avg} values for 50 samples of random networks for a chosen probability, p, which are then used to estimate the mean value of τ_{avg} given by $\bar{\tau}_{avg}$. Then we plot the $\bar{\tau}_{avg}$ values as a function of the rewiring probability in Fig. 4. A curve connecting the data points (blue line in Fig. 4) is used to indicate the trend.

We plot the trend of the average synchronization margin, ρ_{SM}, in Fig. 4 (black markers with a red curve connecting them that indicates the trend). It can be observed, as the value of average τ decreases, the synchronization margin increases, indicating increased randomness (high rewiring probability) in network interconnections makes the network more robust to link uncertainty. It should be noted, there are small variations in the trend shown by ρ_{SM}, which we attribute to the computation of the average value for τ, λ_2, and λ_N for 50 samples of Small World networks. Overall, this trend suggests as the rewiring probability in increased, the network robustness increases. Thus, we conclude nearest neighbour and Small World networks are less robust to the injection of uncertainty at a random link location in the network, compared to random network.

Optimal Gain Result

In this subsection we provide the lemma and proof for the results, which provides a method to design the optimal coupling gain for synchronization.

Lemma 6 *For the network system in Eq.* (6) *with SM given by Eq.* (38)*, the optimal gain,* g^**, to achieve maximum SM is*

$$g^* = \frac{2(a - \frac{1}{\delta})}{\max\{\lambda_N, \lambda_2 + 2\bar{\gamma}\tau\} + \lambda_2 + 2\bar{\gamma}\tau}. \tag{59}$$

Proof. We observe from (40) to maximize the synchronization margin with respect to the coupling gain, g, we must minimize α_0^2 with respect to g, and maximize α_0^2 with respect to λ. This is a regular saddle-point optimization problem [9]. Hence, for a given λ,

$$\frac{\partial \alpha_0^2(\lambda, g)}{\partial g} = -2a_0\lambda + 2\left(\lambda^2 + 2\bar{\gamma}\tau\lambda\right) g = 0. \tag{60}$$

This provides us with optimal gain, and the corresponding α_0^2,

$$g^*(\lambda) = \frac{a_0}{\lambda + 2\bar{\gamma}\tau}, \qquad \text{and,} \qquad \alpha_0^2(\lambda, g^*(\lambda)) = \frac{2\bar{\gamma}\tau a_0^2}{\lambda + 2\bar{\gamma}\tau}. \tag{61}$$

The only important eigenvalues for the graph Laplacian that provide limitations on synchronization (small magnitude of ρ_{SM}) are λ_2 and λ_N. Hence, we obtain,

$$g^*(\lambda_2) = \frac{a_0}{\lambda_2 + 2\bar{\gamma}\tau}, \qquad \text{and,} \qquad g^*(\lambda_N) = \frac{a_0}{\lambda_N + 2\bar{\gamma}\tau}. \tag{62}$$

Since $\lambda_N \geq \lambda_2$, we have

$$g^*(\lambda_2) \geq g^*(\lambda_N) \qquad \text{and} \qquad \alpha_0^2(\lambda_2, g^*(\lambda_2)) \geq \alpha_0^2(\lambda_N, g^*(\lambda_N)). \tag{63}$$

There also exists a value of gain, g_e, which provides the exact same synchronization margin for both λ_2 and λ_N. This is obtained by equating,

$$\alpha_0^2(\lambda_2, g_e) = \alpha_0^2(\lambda_N, g_e), \tag{64}$$

which provides

$$\lambda_2^2 g_e^2 + 2\lambda_2\bar{\gamma}\tau g_e^2 - 2a_0\lambda_2 g_e = \lambda_N^2 g_e^2 + 2\lambda_N\bar{\gamma}\tau g_e^2 - 2a_0\lambda_N g_e. \tag{65}$$

For $\lambda_N \neq \lambda_2$ and $\bar{\lambda} = \frac{\lambda_2 + \lambda_N}{2}$, this gives

$$g_e = \frac{a_0}{\bar{\lambda} + \bar{\gamma}\tau}. \tag{66}$$

Furthermore, the α_0^2 value for g_e, is given by

$$\alpha_0^2(\lambda_2, g_e) = \alpha_0^2(\lambda_N, g_e) = a_0^2 - \frac{4\lambda_2\lambda_N a_0^2}{(\lambda_N + \lambda_2 + 2\bar{\gamma}\tau)^2}. \tag{67}$$

Since $\lambda_N \geq \lambda_2$, we have $g_e \geq g^*(\lambda_N)$. Furthermore,

$$\alpha_0^2(\lambda_N, g_e) \geq \alpha_0^2(\lambda_N, g^*(\lambda_N)) \qquad \text{and} \qquad \alpha_0^2(\lambda_2, g_e) \geq \alpha_0^2(\lambda_2, g^*(\lambda_2)). \tag{68}$$

We also conclude, $g^*(\lambda_2) \geq g_e$, iff $\lambda_N \geq \lambda_2 + 2\bar{\gamma}\tau$ and $g_e \geq g^*(\lambda_2)$, iff $\lambda_2 + 2\bar{\gamma}\tau \geq \lambda_N$. We observe, $\lambda_N \geq \lambda_2 + 2\bar{\gamma}\tau$, iff

$$\alpha_0^2(\lambda_N, g^*(\lambda_2)) \geq \alpha_0^2(\lambda_N, g_e) \geq \alpha_0^2(\lambda_2, g^*(\lambda_2)). \tag{69}$$

Hence, g_e, being the saddle-point solution, is the optimal gain providing the largest possible $\alpha_0^2(\lambda, g)$ and the smallest ρ_{SM}. Similarly, $\lambda_2 + 2\bar{\gamma}\tau \geq \lambda_N$, iff

$$\alpha_0^2(\lambda_2, g_e) \geq \alpha_0^2(\lambda_2, g^*(\lambda_2)) \geq \alpha_0^2(\lambda_N, g^*(\lambda_2)). \tag{70}$$

This gives $g^*(\lambda_2)$ as the optimal gain. Furthermore, at the optimal gain, we always have $\lambda_{sup} = \lambda_2$. Defining, $\chi := \max\{\lambda_N, \lambda_2 + 2\bar{\gamma}\tau\}$, we can write the optimal gain,

$$g^* = \frac{2a_0}{\chi + \lambda_2 + 2\bar{\gamma}\tau}. \tag{71}$$

Hence, for $\lambda_{sup} = \lambda_2$, we obtain

$$\rho_{SM}(g^*) = 1 - \frac{2\bar{\gamma}\tau\lambda_2\,(g^*)^2}{\left(1 - \frac{1}{\delta}\right)^2 - (a_0 - \lambda_2 g^*)^2}. \tag{72}$$

■

We will now provide a lemma which will help readers understand the discussion in this paper on the significance of the Laplacian eigenvalues. For $G(V, \mathcal{E})$ with node set V and edge set, $\mathcal{E}$, let $\mathcal{E}_V$ be all possible connections between nodes in V. Then, for $\tilde{\mathcal{E}} = \mathcal{E}_V \setminus \mathcal{E}$, the graph, $\tilde{G} = (V, \tilde{\mathcal{E}})$, is the compliment of G. Let $0 = \lambda_1 < \lambda_2 \leq \cdots \leq \lambda_N$ be the eigenvalues for G and $0 = \tilde{\lambda}_1 < \tilde{\lambda}_2 \leq \cdots \leq \tilde{\lambda}_N$ be the eigenvalues for $\tilde{G}$. We state below, the lemma connecting the eigenvalues of graph, G, and its complement, $\tilde{G}$ [10].

Lemma 7 *Let $G \equiv (V, \mathcal{E})$ be a graph on $|V| = N$ nodes. Suppose $\tilde{G} \equiv (V, \tilde{\mathcal{E}})$ is the complement of G, such that $\tilde{G} = K_N \backslash G$, where K_N is the complete graph on N vertices. Let $\mathcal{L}_G$ and $\mathcal{L}_{\tilde{G}}$ be the Laplacian matrices of G and $\tilde{G}$ with eigenvalues, $0 = \lambda_1 \leq \lambda_2 \leq \cdots \leq \lambda_N$ and $0 = \tilde{\lambda}_1 \leq \tilde{\lambda}_2 \leq \cdots \leq \tilde{\lambda}_N$, respectively. Then, we must have*

$$\tilde{\lambda}_1 = \lambda_1 = 0,\ \tilde{\lambda}_i = N - \lambda_{N-i+2},\ \forall\, i \in \{2, \ldots, N\}. \tag{73}$$

Simulation Results

In this subsection, we verify the sufficient condition obtained for mean square synchronization through simulation results. We consider the following 1D system,

$$x_{t+1} = a x_t - \phi(x_t) + v_t, \tag{74}$$

where $a = 1.125$, $\delta = 8$, and v_t is additive white Gaussian noise with zero mean and variance ω^2. Here, $\phi(x)$ is given by

$$\phi(x_t) = \frac{\operatorname{sgn}(x_t)}{8}\left(s_1\left(|x_t| - \epsilon\right) + \left(s_2^2(|x_t| - \epsilon)^2 + s_3\right)^{\frac{1}{2}}\right), \tag{75}$$

where $s_1 = 1 + m_2$, $s_2 = 1 - m_2$, $s_3 = 4m_2\epsilon^2$, $m_2 = \frac{1}{1+10\epsilon^{0.1}}$, and $\epsilon = 0.3$. The internal dynamics of the system, as described by Eq. (74), consists of a double-well potential, with an unstable equilibrium point at the origin and two stable equilibrium points at $x^* = \pm\epsilon\left(\frac{a-1}{a-2} + \frac{m_2(a-1)}{m_2(a-1)-1}\right) = \pm 0.5237$. So, with no network coupling, i.e., $g = 0$, the internal dynamics of the agents will converge to the positive equilibrium point, $x^* > 0$, for positive initial conditions. Similarly, if the initial condition is negative, the systems converge to the negative equilibrium point, $x^* < 0$. The double-well potential system is a prototypical example for modeling synchronization phenomena occurring in the natural sciences and engineering systems. For example, collective motion in molecular dynamics [11] and synchronization of generators in the power grid [12] can essentially be modeled using double-well potential.

Effect of coupling gain: We couple this system over a network of 100 nodes, generated as a random network with the Small World property. We choose 60% of the links to be uncertain, making $\tau \approx 1$. The coupling gain for this system is $g = 0.005$. The nominal Laplacian for the network is a standard Laplacian with unit weight. Thus, for all links, e_{ij}, connecting nodes i and j, $\mu_{ij} = 1$. This network has $\lambda_N = 52.55$ and $\lambda_2 = 26.23$. We now choose 50% of the links in the network to have uncertain weights. The uncertainty in the network link weights is chosen as a uniform variable with zero mean and variance, $\sigma^2 = 2$, such that both these eigenvalues satisfy the required condition from the main result. The CoD of the link uncertainty is $\bar{\gamma} = \frac{\sigma^2}{\mu} = 2$. In Fig. 5(a), we plot the results for synchronization of

these 100 systems with simulated additive white Gaussian noise with zero mean and variance, $\omega^2 = 0.1$, which show the systems synchronize in an interval around the equilibrium point.

For systems over the network with identical parameters to those in the previous case and identical link noise variance, if the coupling gain is decreased to $g = 0.001$, which does not satisfy the requirement for the main result, we observe the system is unable to synchronize (Fig. 5(b)).

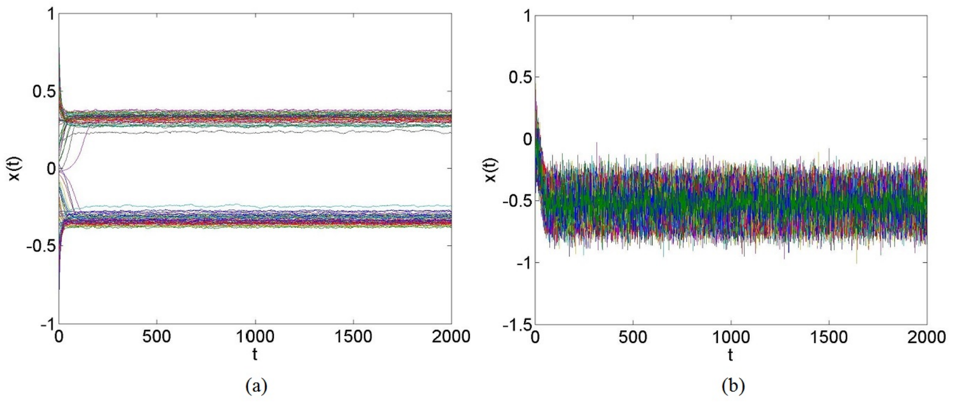

FIG. 5: (a) Time evolution of systems over a 100-node Small World network, $\bar{\gamma} = 2$, $g = 0.01$, with $\rho_{SM} > 0$, (b) time evolution of systems over a 100-node Small World network, $\bar{\gamma} = 2$, $g = 0.001$, with $\rho_{SM} = 0$.

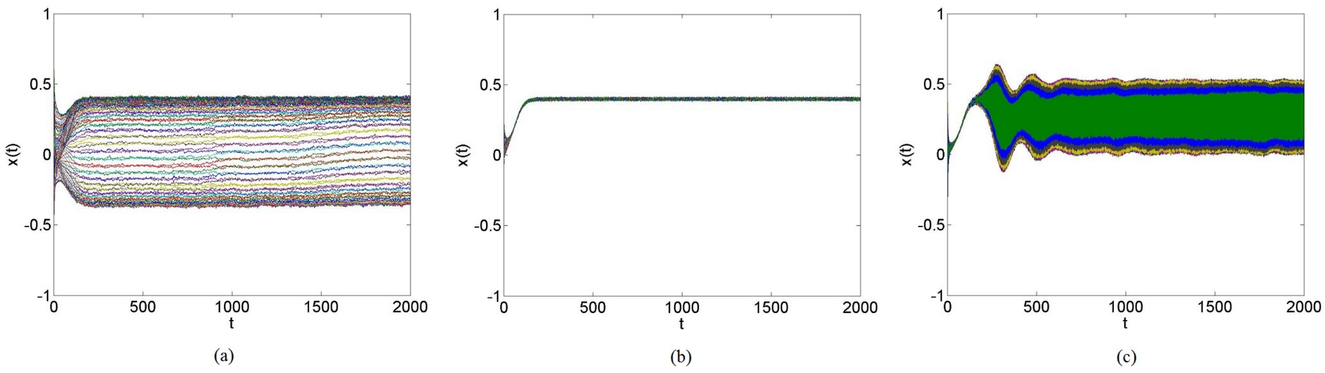

FIG. 6: (a) Time evolution of systems over a 100-node nearest neighbor network with 6 neighbors per agent, $\rho_{SM} = 0$, (b) time evolution of systems over a 100-node nearest neighbor network with 20 neighbors per agent, $\rho_{SM} > 0$, (c) time evolution of systems over a 100-node nearest neighbor network with 32 neighbors per agent, $\rho_{SM} = 0$.

Effect of number of neighbors: Next, we study the effect on the group's synchronization ability, due to a change in the number of neighbors for an agent. For this we choose a nearest neighbor network with 100 nodes. The simulation parameters are chosen as $a = 1.05$, $\delta = 16$, $g = 0.05$, $\gamma = \frac{1}{12}$, and $\tau \approx 1$. The variance for the uncertainty in the links is chosen small, to clearly observe the effects due to a change in neighbors. As discussed previously, increasing the link uncertainty adds to some numerical inaccuracies in the system causing an additive noise-like effect. Furthermore, the additive noise is also assumed absent to facilitate a clear observation of synchronization.

We first choose a network with 6 neighbors per agent. For this network we observe the system is unable to synchronize and all the agents break into multiple clusters with each cluster having a small number of agents, Fig. 6(a). The agents do not obtain sufficient state information to bind them to the synchronization manifold, due to the small number of neighbors. Now, we increase the number of neighbors to 20 for each agent. As the number of neighbors increases, the agents synchronize to the synchronization manifold extremely well, with very little noise, Fig. 6(b). Furthermore, the rate of synchronization is very high as observed from the simulations, where the agents seem to synchronize within the first 100 seconds and then collectively move to the synchronization manifold. Synchronization

of the agents is observed for a number of neighbors starting at 16 until the number of neighbors reaches 28. As the number of neighbors increases, we observe significant oscillations before the agents synchronize. Finally, we increase the number of neighbors to 32 for each agent. This increase in the number of neighbors seems to benefit the synchronization initially, since all agents quickly coalesce together. However, as they approach the synchronization manifold, the high number of neighbors causes the systems to fluctuate significantly about the manifold, leading to an oscillating band of desynchronized agent states, Fig. 6(c). This is the beginning of a desynchronized state for the agents. More neighbors for an agent would destabilize the individual system dynamics.

---

[1] Has'minskiĭ, R. Z. *Stability of differential equations.* Sijthoff & Noordhoff, Germantown ,MD, (1980).

[2] Wang, Z., Wang, Y., & Liu, Y. Global synchronization for discrete-time stochastic complex networks with randomly occurred nonlinearities and mixed time delays. *IEEE Trans. Neural Netw.*, **21**, 11–25(2010).

[3] Haddad, W., & Bernstein, D. Explicit construction of quadratic Lyapunov functions for the small gain theorem, positivity, circle and Popov theorems and their application to robust stability. Part II: Discrete-time theory. *Int. J. Robust Nonlin.*, **4**, 249–265(1994).

[4] Lancaster, P., & Rodman, L. *Algebraic Riccati Equations.* Oxford Science Publications, Oxford, (1995).

[5] Astrom, K. J., & Murray, R. M. *Feedback Systems: An Introduction for Scientists and Engineers.* Princeton University Press, Princeton, NJ, (2008).

[6] Dullerud, G. E., & Paganini, F. *A Course in Robust Control Theory.* Springer-Verlag, New York, NY, (2000).

[7] Elia, N, Remote Stabilization over Fading Channels. *Syst. Control Lett.*, **54**, 237-249(2005)

[8] Watts, D. J., & Strogatz, S. H. Collective dynamics of small-world networks. *Nature*, **393**, 409–10(1998).

[9] Boyd, S., & Vandenberghe, L. *Convex Optimization.* Cambridge University Press, Cambridge, UK, (2003).

[10] Merris, R. Laplacian matrices of graphs: a survey. *Linear Algebra Appl.*, **197-198**, 143 – 176(1994).

[11] Mezic, I. On the dynamics of molecular conformation. *Proc. Natl. Acad. Sci. U.S.A.*, **103**, 7542–7547(2006).

[12] Dörfler, F., Chertkov, M., & Bullo, F. Synchronization in complex oscillator networks and smart grids. *Proc. Natl. Acad. Sci. U.S.A.*, (2013).

[13] Not to be confused with stochastic or uncertain interactions in the network. While the *random* in the small world network has to do with random generation of network topology which is static in time, the uncertain network interactions which is the main focus of this paper has to do with dynamic stochastic interactions between network nodes.
